# HNF1B, EZH2 and ECI2 in prostate carcinoma. Molecular, immunohistochemical and clinico-pathological study

**DOI:** 10.1038/s41598-020-71427-7

**Published:** 2020-09-01

**Authors:** Pavel Dundr, Michaela Bártů, Jan Hojný, Romana Michálková, Nikola Hájková, Ivana Stružinská, Eva Krkavcová, Ladislav Hadravský, Lenka Kleissnerová, Jana Kopejsková, Bui Quang Hiep, Kristýna Němejcová, Radek Jakša, Otakar Čapoun, Jakub Řezáč, Kateřina Jirsová, Věra Franková

**Affiliations:** 1 Institute of Pathology, First Faculty of Medicine, Charles University and General University Hospital in Prague, Studničkova 2, 12800 Prague 2, Czech Republic; 2 Institute of Pathology, First Faculty of Medicine, Charles University, Prague 2, Czech Republic; 3 Department of Urology, First Faculty of Medicine, Charles University and General University Hospital in Prague, Prague 2, Czech Republic; 4 Institute of Biology and Medical Genetics, First Faculty of Medicine, Charles University and General University Hospital in Prague, Prague 2, Czech Republic; 5 Department of Pediatrics and Adolescent Medicine, First Faculty of Medicine, Charles University and General University Hospital in Prague, Prague 2, Czech Republic

**Keywords:** Cancer, Molecular biology

## Abstract

Hepatocyte nuclear factor 1 beta (HNF1B) is a tissue specific transcription factor, which seems to play an important role in the carcinogenesis of several tumors. In our study we focused on analyzing HNF1B in prostate carcinoma (PC) and adenomyomatous hyperplasia (AH), as well as its possible relation to the upstream gene *EZH2* and downstream gene *ECI2*. The results of our study showed that on an immunohistochemical level, the expression of HNF1B was low in PC, did not differ between PC and AH, and did not correlate with any clinical outcomes. In PC, mutations of *HNF1B* gene were rare, but the methylation of its promotor was a common finding and was positively correlated with Gleason score and stage. The relationship between HNF1B and EZH2/ECI2 was equivocal, but EZH2 and ECI2 were positively correlated on both mRNA and protein level. The expression of EZH2 was associated with poor prognosis. ECI2 did not correlate with any clinical outcomes. Our results support the oncosuppressive role of HNF1B in PC, which may be silenced by promotor methylation and other mechanisms, but not by gene mutation. The high expression of EZH2 (especially) and ECI2 in PC seems to be a potential therapeutic target.

## Introduction

Hepatocyte nuclear factor 1 beta (HNF1B) is an important tissue specific transcription factor, which is essential for the embryonic development of several organs including the kidney, pancreas, liver, biliary tract, genital tract, and gastrointestinal system^1,2^. Heterozygous germline mutations are associated with several congenital diseases of the kidney, pancreas, and urogenital tract, and the role of HNF1B in these diseases is well known^3–5^. However, despite the increased awareness that HNF1B plays an important role in the carcinogenesis of several solid tumors, the exact significance of HNF1B in carcinogenesis is not yet fully understood^6–9^. The regulatory mechanisms and pathways in which HNF1B is involved in the process of carcinogenesis are not clear, but it appears that HNF1B may act as either oncogene or oncosuppressor gene based on the type of tumor and its histogenesis^6,7,10^.

In the prostate, an increased expression of HNF1B seems to be protective against prostate cancer, and HNF1B therefore has been attributed an oncosuppressive role^10^. However, the levels of protein expression of HNF1B in prostate cancer are not well known, and the mechanisms playing role in its regulation are of interest. According to current knowledge, HNF1B is commonly inactivated in prostate cancer, especially due to *HNF1B* promoter methylation, which occurs in about 50% of cases^11^. Another recently suggested possible mechanisms of *HNF1B* inactivation is the effect of the enhancer of zeste homolog 2 (*EZH2*), the overexpression of which has been suggested to downregulate the expression of *HNF1B*^12,13^. *EZH2* is a gene belonging to the category of Polycomb Group (*PcG*) genes, and the EZH2 protein is a part of the multiproteic complex Polycomb Repressive Complex 2 (PRC2)^14^. This complex plays a critical role during embryogenesis via mediating epigenetic gene silencing. The PRC2 complex is responsible for the methylation of lysine 27 of histone H3 via enzymatic subunits enhancer of zeste homolog 1 (EZH1) and EZH2. The expression of PRC2 proteins is upregulated in several tumors, including prostate carcinoma^15^. Based on functional studies, EZH2 seems to be act as an oncogene^16^. EZH2 protein overexpression has been found in hormone-refractory metastatic prostate cancer^15^. Moreover, overexpression of EZH2 in localized prostate carcinoma is a sign of an adverse prognosis^13,15,17^. EZH2 promotes increased cell proliferation leading to prostate cancer progression, and is associated with increased metastatic capability by promoting epithelial-mesenchymal transition (EMT)^13,15,18–22^. Except for its function as a transcriptional repressor, EZH2 can also directly react with androgen receptor (AR) and act as its co-activator^23,24^. Also, an association between the overexpression of EZH2 and neuroendocrine differentiation in prostate carcinoma, associated with adverse prognosis, has been described^25^. Inhibition of EZH2 suppresses cancer cell proliferation and invasion, and as such EZH2 seems to be a potential therapeutic target^26–28^.

Concerning downstream effect, HNF1B also seems to be involved in several key regulatory pathways including cell cycle regulation, epithelial-mesenchymal transition, cell migration, adhesion, and proliferation^1,18,29^. HNF1B also plays a role in glucose metabolism, influencing both insulin secretion and renal glucose reabsorption, which is one of the key mechanisms in selective advantage of several tumor types^30^. Recently, it has been shown in an animal model that the downregulation of HNF1B protein levels during tumor progression is associated with the upregulation of enoyl-CoA-(Δ) isomerase 2 (ECI2), which is one of the possible downstream targets of HNF1B^31^.

Both HNF1B and ECI2 are assumed to be downstream targets of AR, which is a major driver in prostate cancer^31,32^. However, the specific interaction between HNF1B and ECI2 is uncertain, but according to recent data, the increased gene expression of *ECI2* may promote prostate cancer growth^32^. ECI2 belongs to the family of acyl-CoA-binding domain (ACBD) together with enoyl-CoA-(Δ) isomerase 1 (ECI1). Their role in β-oxidation is to catalyze the conversion of different combinations of double bonds of coenzyme A (CoA)-bound fatty acids to trans-2 configuration, allowing for the re-entrance of enoyl-CoA into the β-oxidation cycle^33^. ECI2 is localized in both the mitochondria and peroxisomes, whereas ECI1 is specific to mitochondria only^34^. It has been suggested that acyl-CoA-binding domain (ACBD2)/ECI2 mediate the direct contact between peroxisomes and mitochondria, which is, in mammals, still poorly understood. This interaction seems to be an important part of metabolite exchange during steroid hormone biosynthesis^35^. ECI2 also plays a role in glucose metabolism, where it increases glucose consumption and lactate production^32^. The exact role of ECI2 in the process of carcinogenesis is not well known. However, it has been shown that peroxisomes play a crucial role in mammalian metabolism and may play a role in the process of carcinogenesis^36–39^. In an animal model, it has been shown that a high-fat diet (or diet rich in the so-called “peroxisome proliferators” such as hypolipidemic fibric acid derivatives) results in the proliferation of peroxisomes, especially in the liver, and in a lesser amount in other tissues such as the kidney, heart and intestinal mucosa^39,40^. Finally, this peroxisomal proliferation was associated with development of liver tumors^37^. The mechanism of this effect is not yet entirely clear, but it has been suggested that the tumorigenic potential is associated with the stimulation of genes encoding enzymes of the β-oxidation system, which may play a role in the carcinogenesis. The mechanism of liver carcinogenesis may be also associated with the fact that ECI1 plays an important role in HCV pathogenesis^41^.

The goal of our study was to perform a comprehensive analysis of the protein and mRNA expression, epigenetic and genetic changes of HNF1B in prostate carcinoma (PC), focusing also on its relationship with the possible upstream (*EZH2*) and downstream (*ECI2*) genes on the level of protein expression, as detected by immunohistochemistry and mRNA expression. The results were correlated with clinico-pathological variables and clinical outcomes. A small sample set of adenomyomatous hyperplasia (AH) was used as a control group.

## Results

The immunohistochemical analysis of all three markers including HNF1B, EZH2 and ECI2 was performed on the total of 101 PC and 18 AH samples. The analysis of immunohistochemical expression of AR was performed on 96 PC samples.

Mutation analysis of *HNF1B* was performed on 77 samples of PC and 14 samples of AH. Due to the requirement of high-quality genetic material, it was possible to carry out the DNA methylation analysis and mRNA expression analysis (HNF1B, EZH2, ECI2) only on the frozen tissue (FT) samples (including 54 PT and 14 AH).

### Immunohistochemical findings

**Figure 1:**
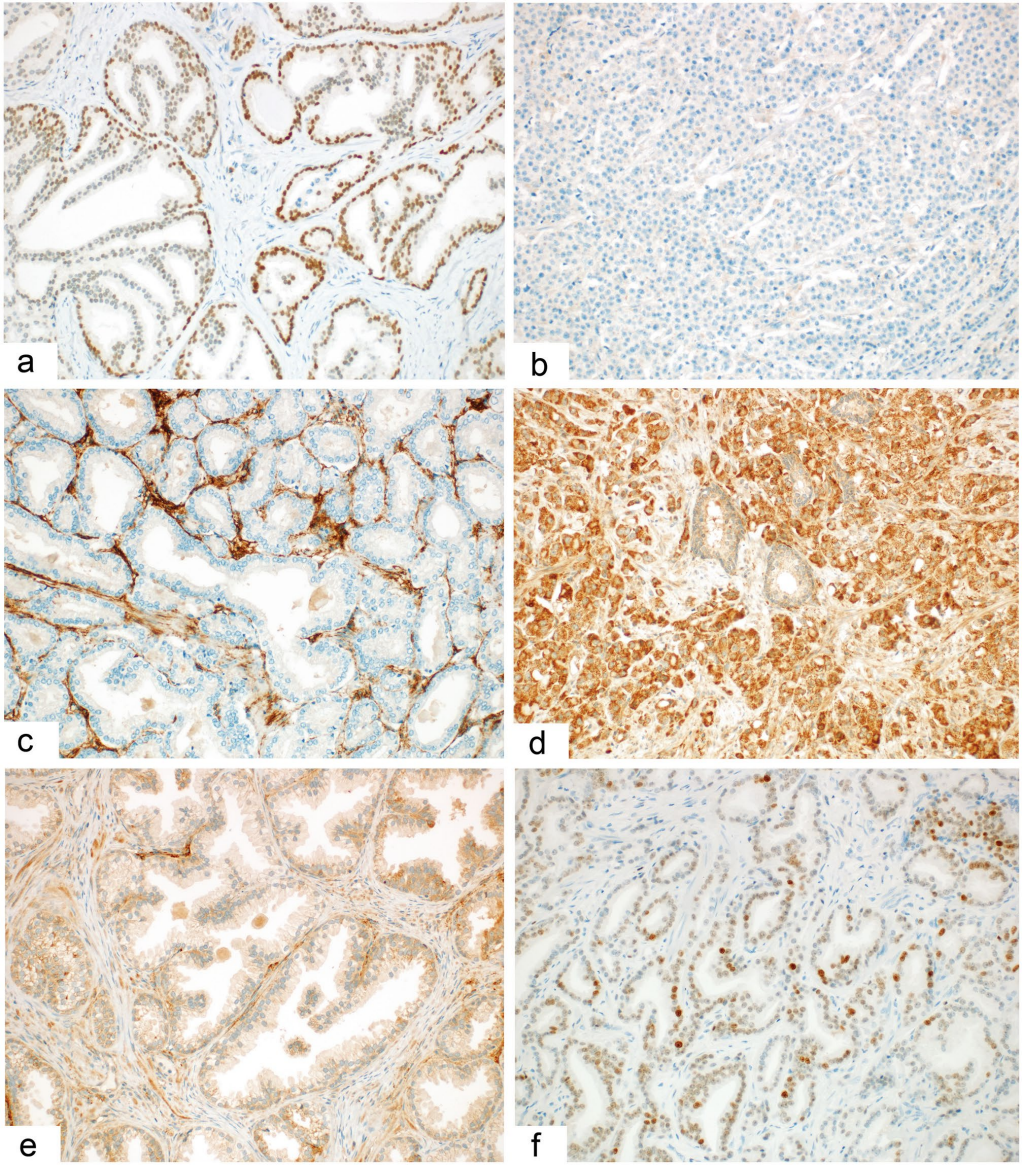
Representative examples of the immunohistochemical results. Immunohistochemical expression of HNF1B in prostate carcinoma showing variable intensity of positivity in most of the tumor cells **(A)** (× 200) and a complete negativity in a poorly differentiated (Gleason score 9) carcinoma **(B)** (× 200). Immunohistochemical expression of ECI2 with complete negativity in tumor cells and positivity in the stroma **(C)** (× 200); diffuse positivity in a poorly differentiated carcinoma **(D)** (× 200); and a weak positivity in an adenomyomatous hyperplasia sample **(E)** (× 200). Immunohistochemical expression of EZH2 in prostate carcinoma showing variable intensity of positivity of some of the tumor cells **(F)** (× 200).

**Figure 2:**
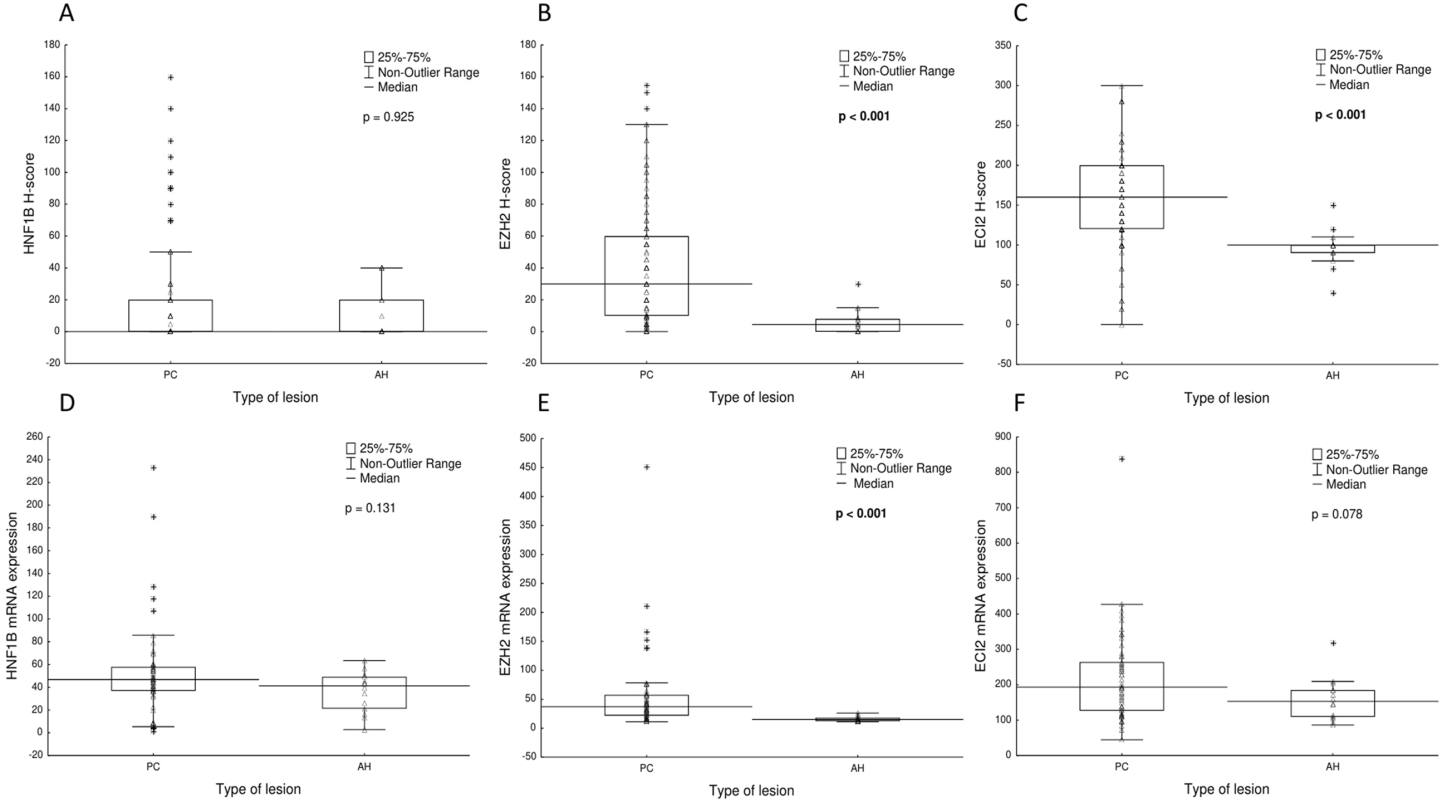
Comparison of HNF1B, EZH2 and ECI2 expression on a protein **(A–C)** and mRNA level **(D–F)** in the prostate cancer (PC, N = 101) and adenomyomatous hyperplasia (AH, N = 18) samples. Significant p-values are indicated in bold.

**Figure 3:**
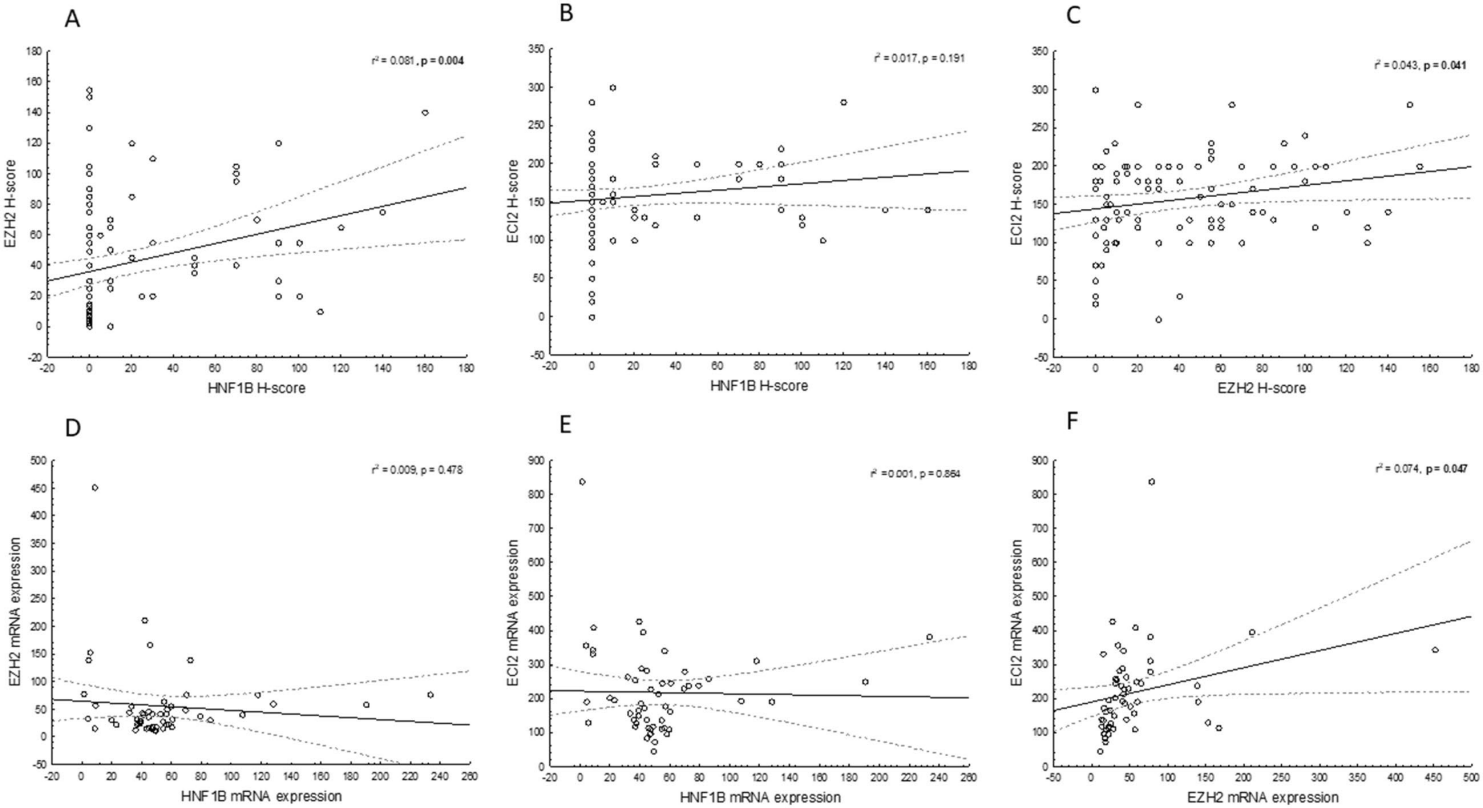
Correlation of HNF1B, EZH2 and ECI2 expression on a protein **(A–C)** and mRNA **(D–F)** level, based on 54 cases of prostate cancer. Significant p-values are indicated in bold. Dashed lines represent 0.95 confidence intervals.

All immunohistochemical findings are summarized in Tables 1 and 2. Representative examples are shown in Fig. 1. The comparison of protein/mRNA expression of all the markers in prostate lesions (PC and AH) is shown in Fig. 2. The raw data is available in Supplementary Table 1. Briefly, the protein expression of HNF1B in both PC and AH was very low, with a mean H-score of 19.5 for PC and 11.7 for AH. The differences between PC and AH were not significant (U = 880, Z = − 0.08, p = 0.925). The protein expression of EZH2 and ECI2 was significantly higher in PC than in AH samples (EZH2: U = 305.5, Z = 4.42; ECI2: U = 251.5, Z = 4.83, p < 0.001). Data for the AH group is however limited and unbalanced, as the sample size is only 18 cases. The protein expression of AR was analyzed for the PC group only, with the following results: mean = 126.7 ± 90.8, median = 100, N = 96. There was a statistically significant positive correlation between the protein expression of HNF1B and EZH2 (F = 8.52, p = 0.004) and a statistically insignificant positive correlation between the expression of HNF1B and ECI2 (F = 1.7, p = 0.191). The positive correlation between the H-score of EZH and ECI2 reached marginal significance (F = 4.30, p = 0.041). The relationships between protein expressions on an immunohistochemical level are shown in Fig. 3A–C.

**Table 1:** Association of the HNF1B/EZH2/ECI2 H-score with the clinico-pathological variables, based on 101 patients with prostate carcinoma.

Characteristic	Group	N	HNF1B	HNF1B	p-value	EZH2	EZH2	p-value	ECI2	ECI2	p-value
			mean ± SD	median		mean ± SD	median		mean ± SD	median	
**Age**					0.728			0.938			0.871
	˂ 66	53	17.8 ± 35.3	0		43.5 ± 42.6	30		153.4 ± 61.6	160	
	≥ 66	48	21.4 ± 38.3	0		39.8 ± 36.2	30		160.4 ± 55.9	160	
**Staging**					0.871			**0.009**			0.118
	T1 + 2	49	22.2 ± 41.0	0		32.4 ± 35.4	20		145.5 ± 53.0	140	
	T3 + 4	52	16.6 ± 32.0	0		50.7 ± 41.6	49		165.0 ± 62.6	180	
**Gleason score**					0.943			**0.007**			0.491
	Low	16	15.0 ± 25.8	0		29.6 ± 37.6	16		156.2 ± 55.1	170	
	Intermediate	58	20.1 ± 39.3	0		35.6 ± 36.3	20		147.0 ± 58.6	140	
	High	27	20.9 ± 38.1	0		60.7 ± 42.7	55		169.2 ± 61.7	170	
**Resection margin**					0.887			0.439			0.357
	R0	67	18.7 ± 34.2	0		38.9 ± 37.1	30		159.5 ± 61.2	170	
	R1	34	21.1 ± 42.5	0		47.9 ± 44.3	45		150.6 ± 53.9	145	
**Lymphovascular invasion**					0.229			0.083			0.267
	Yes	20	24.0 ± 37.0	2		55.6 ± 42.8	55		172.5 ± 55.7	175	
	No	81	18.4 ± 37.1	0		38.3 ± 38.3	30		152.6 ± 59.2	150	
**Perineural invasion**					0.163			0.498			0.107
	Yes	86	21.4 ± 38.0	0		42.9 ± 39.6	30		160.5 ± 55.6	165	
	No	15	8.6 ± 28.5	0		36.0 ± 40.4	30		135.3 ± 72.7	120	
Metastases*					0.824			**0.023**			0.867
	Yes	4	17.5 ± 35.0	0		81.3 ± 16.5	83		160.0 ± 36.5	160	
	No	93	19.1 ± 37.3	0		39.6 ± 39.7	30		154.9 ± 77.1	150	
Preoperative PSA level (ng/mL)*					0.293			0.077			0.959
	≤ 4	8	23.8 ± 38.9	5		56.8 ± 54.3	40		160 ± 59.5	140	
	4.1–10	54	14.4 ± 34.6	0		34.0 ± 37.2	20		155.4 ± 56.2	155	
	10.1–20	10	20.0 ± 27.5	5		58.0 ± 43.4	60		158 ± 48.0	165	
	> 20	11	27.3 ± 49.4	0		60.9 ± 43.8	55		147.3 ± 66.3	150	
Methylation*					0.335			NA			NA
	Yes	28	11.2 ± 26.8	0		NA	NA		NA	NA	
	No	24	30.0 ± 51.4	0		NA	NA		NA	NA	
rs4430796*					0.969			NA			NA
	Yes	44	19.4 ± 41.1	0		NA	NA		NA	NA	
	No	8	22.5 ± 41.6	0		NA	NA		NA	NA	
Biochemical recurrence*					0.373			**0.003**			0.856
	Yes	20	26.1 ± 42.1	0		62.9 ± 37.4	55		160.5 ± 38.7	160	
	No	75	17.1 ± 36.0	0		35.8 ± 39.3	20		155.8 ± 62.9	150	

Significant p-values are indicated in bold.

*Data not available for all cases.

**Table 2:** Association of the HNF1B/EZH2/ECI2 H-score with the type of lesion, based on 119 patients with prostate lesion. Significant p-values are indicated in bold.

Type of lesion	N	HNF1B mean ± SD	HNF1B median	p-value	EZH2 mean ± SD	EZH2 median	p-value	ECI2 mean ± SD	ECI2 median	p-value
				0.925			** < 0.001**			** < 0.001**
PC	101	19.5 ± 36.6	0		41.8 ± 39.6	30		156.6 ± 58.8	160	
AH	18	11.7 ± 16.9	0		5.9 ± 7.7	4.5		96.1 ± 21.7	100	

*PC* prostate cancer, *AH *adenomyomatous hyperplasia.

The results of EZH2, HNF1B and ECI2 expression were correlated with the immunohistochemical expression of AR. We have found a significant, positive correlation between EZH2 and AR expression (F = 42.5, p < 0.001), HNF1B and AR expression (F = 36.3, p < 0.001), and ECI2 and AR expression (F = 9.95, p = 0.002).

### Expression of mRNA

The results of mRNA expression in PC and AH are summarized in Table 3. Briefly, the expression of HNF1B and ECI2 mRNA was not statistically significantly different between the carcinoma and hyperplasia group (HNF1B: U = 278.0, Z = 1.51, p = 0.131; ECI2: U = 261.0, Z = 1.77, p = 0.078), although both genes showed a lower expression in AH (Fig. 2D, F). The expression of EZH2 mRNA was statistically significantly higher in the PC group when compared to the AH group (U = 96.0, Z = 4.27, p < 0,001; Fig. 2E).

**Table 3:** Association of the mRNA expression of HNF1B/EZH2/ECI2 with the type of lesion, based on 68 patients with prostate lesion. Significant p-values are indicated in bold.

Type of lesion	N	HNF1B mean ± SD	HNF1B median	p-value	EZH2 mean ± SD	EZH2 median	p-value	ECI2 mean ± SD	ECI2 median	p-value
				0.131			** < 0.001**			0.078
PC	54	53.1 ± 40.8	46.7		55.4 ± 68.5	37.2		217.6 ± 127.7	193.2	
AH	14	36.1 ± 17.7	41.2		16.2 ± 4.6	15.2		158.8 ± 61.3	152.7	

Concerning the relationship between HNF1B and EZH2 mRNA expression in the PC group, there was an apparent negative trend, which, however, was not significant in our dataset (F = 0.51, p = 0.478) (Fig. 3D). The expression of both HNF1B and ECI2 shows a similar, weak negative trend (F = 0.03, p = 0.864) (Fig. 3E). There was also a marginally significant positive correlation between ECI2 and EZH2 mRNA expression (F = 4.15, p = 0.047, Fig. 3F).We observed a positive correlation between the mRNA expression and protein expression for each marker in the PC group. An increased protein expression correlated with increased mRNA levels, but only the data for EZH2 reached statistical significance (F = 4.55, p = 0.037), compared to HNF1B (F = 1.88, p = 0.175) and ECI2 (F = 1.38, p = 0.245).

### Methylation analysis

Methylation analysis was successfully performed in 53 PC and 16 AH samples. The methylation of the *HNF1B* promoter was more commonly observed in PC than in AH (χ^2^ = 11.75, p < 0.001). Methylation was found in 29/53 carcinoma (55%), and 1/16 hyperplasia (6%) samples. No significant correlation between promoter methylation status (methylated – N = 28 × non-methylated – N = 24) and HNF1B expression based on either IHC (U = 283.0, Z = − 0.96, p = 0.217) or mRNA expression (U = 299.0, Z = − 0.67, p = 0.502) was found.

### Sequencing analysis

Mutation analysis of *HNF1B* was successfully performed in 77 PC and 14 AH samples. In one PC case, a single somatic variant of uncertain significance was found (NM_000458.2: c.232G > C p.E78Q, mutant allele frequency = 6.4%).

In the FT sample subset only, the intronic polymorphism rs4430796 (G/A) was analyzed by amplicon NGS approach. The frequency of homozygotes GG (15.1% PC; 14.3% AH), AA (24.5% PC; 21.4% AH) and heterozygotes AG (60.4% PC; 64.3% AH) was similar in both of the examined subsets, as well as the overall frequency of alleles G/A (A: 53.2%; G: 46.8%), which is in concordance with the overall population data available in the database 1000Genomes (https://www.internationalgenome.org/; A: 55.4%, G: 44.6%).

### Clinico-pathological correlations

The expression of HNF1B, based on either IHC or mRNA, showed no statistically significant correlation with any of the monitored outcomes (Tables 1 and 5). *HNF1B* promoter methylation was significantly higher among the carcinoma cases of a higher stage (T3 + 4) in comparison to the lower stage (T1 + 2), and also with the higher Gleason score (intermediate and high grade) in comparison to the lower grades (Table 4).

**Table 4:** Association of the HNF1B methylation status with the clinico-pathological characteristics, based on 53 cases of prostate cancer.

Characteristics	Methylated	Non-methylated	p-value
**Age**			0.63
˂ 66	15	14	
≥ 66	14	10	
**Staging**			**0.002**
T1 + 2	6	15	
T3 + 4	23	9	
**Gleason score**			**0.034**
Low grade	1	4	
Medium grade	18	18	
High grade	10	2	
**Resection margin**			0.858
R0	20	16	
R1	9	8	
**Lymphovascular invasion**			0.127
Yes	7	2	
No	22	22	
**Perineural invasion**			0.532
Yes	25	22	
No	4	2	
**Preoperative PSA level (ng/mL)**			0.989
≤ 4	2	2	
4.1–10	20	16	
10.1–20	4	3	
> 20	3	3	
**rs4430796**			0.631
Yes	24	21	
No	5	3	
Biochemical recurrence*			0.917
Yes	5	4	
No	22	19	

Significant p-values are indicated in bold.

*Data not available for all cases.

The increased expression of EZH2 detected by immunohistochemistry (H-score) showed statistically significant positive correlations with stage, Gleason score, metastases, and biochemical recurrence. The increased expression of EZH2 mRNA showed statistically significant positive correlations with stage, Gleason score, and LVSI. In contrast, on the mRNA level, the correlation with metastases and biochemical recurrence (BCR) was not statistically significant.

The expression of ECI2 was associated with stage, but only on the mRNA level (lower mRNA expression in group T1 + T2 when compared to T3 + T4). No significant correlation was detected between ECI2 as evaluated by IHC and the clinico-pathological variables.

**Figure 4:**
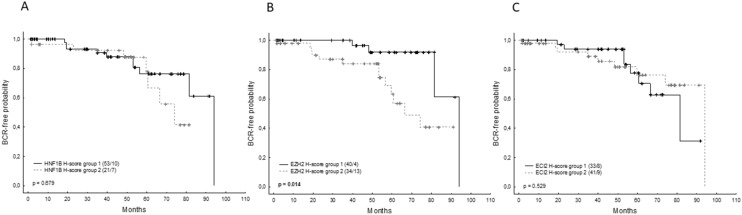
Kaplan–Meier curves for biochemical recurrence (BCR)-free survival in 91 patients according to the H-score of HNF1B (**A**, group 1 = H-score < 10, group 2 = H-score ≥ 10), EZH2 (**B**, group 1 = H-score < 30, group 2 = H-score ≥ 30) and ECI2 (**C**, group 1 = H-score < 150, group 2 = H-score ≥ 150). Censored/complete cases are reported in parentheses. Significant p-values are indicated in bold.

Using a PSA cut-off of 0.2 ng/ml, 16.6% of patients (15/90) experienced biochemical recurrence after surgery after a median follow-up of 53 months (range 2–94). Significant variables associated with biochemical recurrence in the univariate model included pre-operative PSA level (p = 0.001), positive resection margins (p = 0.022), pathological Gleason score (p = 0.012), LVSI (p = 0.045) and EZH2 H-score (p = 0.014) (Fig. 4B). In the multivariate analysis, the pre-operative resection margin (p = 0.012), PSA level (0.022), and EZH2 H-score (p = 0.033) remained significant in the minimal adequate model. Neither the HNF1B H-score, nor the ECI2 H-score were associated with the probability of biochemical recurrence (Fig. 4A, C). The clinico-pathological variables in relation to the mRNA expression of all the analyzed genes are summarized in Table 5.

**Table 5:** Associations of the HNF1B/EZH2/ECI2 mRNA expression with the clinico-pathological variables in a cohort of 54 patients with prostate carcinoma.

Characteristic	Group	N	HNF1B mean ± SD	HNF1B median	p-value	EZH2 mean ± SD	EZH2 median	p-value	ECI2 mean ± SD	ECI2 median	p-value
**Age**					0.532			0.722			0.848
	˂ 66	29	48.1 ± 30.7	44.7		56.8 ± 82.6	40.1		211.7 ± 98.2	194.3	
	≥ 66	25	58.9 ± 50.1	48.7		53.9 ± 49.9	31.3		224.5 ± 157.1	176.9	
**Stage**					0.624			** < 0.001**			**0.030**
	T1 + T2	23	52.7 ± 38.0	48.7		29.8 ± 18.3	22.3		198.4 ± 162.5	138.2	
	T3 + T4	31	53.4 ± 43.3	44.5		74.5 ± 85.1	43.1		231.8 ± 94.7	228.6	
**Gleason score**					0.218			**0.003**			0.182
	Low	5	81.8 ± 61.2	56.2		63.8 ± 60.8	56.1		144.2 ± 59.9	113.2	
	Intermediate	37	47.9 ± 23.8	46.6		39.5 ± 37.6	30.2		222.8 ± 142.9	202.9	
	High	12	57.4 ± 65.9	42.5		101.5 ± 116.9	62		232.2 ± 87.9	191.5	
**Resection margin**					0.391			0.688			0.837
	R0	37	51.7 ± 44.9	45.6		63.8 ± 81.2	40.1		214.3 ± 107.4	202.9	
	R1	17	56.2 ± 31.1	54.9		38.7 ± 20.9	32.4		224.9 ± 167.5	190.9	
**Lymphovascular invasion**					0.944			**0.050**			0.816
	Yes	9	50.0 ± 38.3	47.1		61.5 ± 38.6	55.4		265.4 ± 226.7	190.9	
	No	45	53.7 ± 41.7	46.5		54.3 ± 73.7	31.3		208.1 ± 98.8	194.3	
**Perineural invasion**					0.449			0.591			0.253
	Yes	48	52.9 ± 36.8	47.1		55.5 ± 71.6	37.2		214.1 ± 132.3	188.9	
	No	6	54.6 ± 69.8	31.5		55.4 ± 44.5	44.4		245.5 ± 85.6	224.6	
Metastases*					0.536			0.07			0.872
	Yes	2	30.1 ± 35.0	30.1		108.5 ± 62.6	108.5		186.9 ± 82.0	186.9	
	No	52	54.0 ± 41.0	46.8		53.4 ± 68.8	35.1		218.8 ± 129.6	193.2	
Preoperative PSA level (ng/mL)*					0.611			0.803			0.873
	≤ 4	4	73.2 ± 108.5	27.4		61.7 ± 59.3	46.5		250.4 ± 130.1	261.8	
	4.1–10	38	51.9 ± 33.5	47.2		58.2 ± 79.7	32.5		217 ± 139.3	185.6	
	10.1–20	7	50.5 ± 15.1	54.9		46.1 ± 20.9	45.4		192.2 ± 79.2	196.7	
	> 20	5	50.2 ± 45.3	36.8		42.8 ± 16.2	43.1		231.8 ± 110.4	190.9	
Methylation*					0.502			NA			NA
	Yes	28	57.6 ± 53.7	43.1		NA	NA		NA	NA	
	No	24	49.8 ± 17.3	47.2		NA	NA		NA	NA	
rs4430796*					0.269			NA			NA
	Yes	44	57.2 ± 43.1	46.8		NA	NA		NA	NA	
	No	8	36.7 ± 20.2	43.1		NA	NA		NA	NA	
Biochemical recurrence*					0.301			0.153			0.899
	Yes	5	55.9 ± 39.2	46.7		62.2 ± 38.5	43.1		179.5 ± 72.9	186.7	
	No	46	60.5 ± 40.5	51.7		53.3 ± 43.5	33.0		215.0 ± 136.1	195.5	

Significant p-values are indicated in bold.

*Data not available for all cases.

### Re-analysis of the prostate carcinoma samples TCGA dataset

Only one missense mutation of the *HNF1B* gene was reported in TCGA dataset (NM_00458.4: c.853G > A, p.G285S). This mutation is reported in the ClinVar database as likely pathogenic. Methylation of the *HNF1B* promoter was found in 231/491 (47%) prostate carcinoma samples. There was a significantly lower expression of HNF1B mRNA in the methylated samples (N = 231) in comparison to the non-methylated samples (N = 260; U = 7,534, Z = − 14.34, p < 0.001). A decreasing HNF1B mRNA expression was observed with an increasing Gleason score, and the opposite trend was observed for EZH2 mRNA expression. Furthermore, the increased expression of EZH2 mRNA was observed in cases with stage T3, when compared to stages T1 and T2 (Supplementary Table 2). There was a statistically significant negative correlation between the expression of HNF1B mRNA, and the expression of EZH2 (F = 5.39, p = 0.021) and ECI2 mRNA (F = 4.82, p = 0.028). The expression of ECI2 mRNA and EZH2 mRNA showed a statistically significant positive correlation (F = 13.98, p ˂ 0.001).

## Discussion

The evaluation of the significance of HNF1B in the carcinogenesis of solid tumors has recently been gaining importance, along with the data gained from several genome-wide association studies (GWAS), suggesting that certain HNF1B single nucleotide polymorphisms (SNPs) are associated with either an increased or decreased risk of several solid tumors (such as ovarian carcinoma, endometrial carcinoma and prostate carcinoma)^42–46^. A recent meta-analysis of 18 studies, involving almost 35 thousand patients and 56 thousand controls, showed that there is a strong association between the risk of prostate cancer and single nucleotide polymorphisms rs4430796; rs11649743; rs7501939; and rs3760511^47^. In our study, the rs4430796 (G > A) was present comparably in 85% of patients from the PC group (45/53) and 86% from the AH group (12/14) in the homo- or heterozygous variant. The overall detected ratio of allele frequencies (A: 53.2%; G: 46.8%) virtually corresponds to the global population ratio of this polymorphism (based on 1000G data; A: 55.4%; G: 44.6%), and therefore our dataset did not show any association of this polymorphism with prostate cancer. The increase of risk variant polymorphisms seem to be associated with promoter hypermethylation and decreased HNF1B expression^10^. However, some studies report contradictory results, showing that rs4430796 is associated with a decreased risk of prostate carcinoma^45,48^. In one study, the rs4430796 was associated with an increased mRNA HNF1B expression^49^.

Despite the fact that the involvement of HNF1B in prostate carcinogenesis is a complex and still unresolved issue with some controversies, according to current knowledge and most of the studies published, the increased expression of HNF1B may protect against prostate cancer, suggesting that in the prostate HNF1B seems to have an oncosuppressive role^7,10^. Silencing of the *HNF1B* gene by methylation of its promoter seems to be an important mechanism of *HNF1B* inactivation, which occurs in about 50% of prostate carcinomas^11,12,50^. In our study, we have detected methylation in 55% (29/53 PC cases), which is similar to the data from TCGA (47% of methylated cases)^11^. On the contrary, in AH samples the *HNF1B* promoter methylation occurs in only 1/16 cases (6%). Interestingly, in contrast to TCGA data, we did not find any association between *HNF1B* promoter methylation and mRNA expression, which can be caused by different methodological approach utilized in the obtaining of TCGA data (ChIP-seq analysis of promoter methylation and RNA-Seq analysis of the mRNA expression). The TCGA sample set was significantly larger, although their methylation ChIP-seq analysis may potentially benefit from scaling the methylation level. Our HRM analysis of promoter methylation showed similar results as the ones reported in TCGA. The mRNA expression levels in TCGA were obtained using RNA-Seq approach, which is significantly less sensitive than our ddPCR approach. Moreover, we have found that protein expression did not correlate with methylation levels, which indicates that another mechanism may be involved in regulating HNF1B expression. The data about protein expression is not available in TCGA.

Except for methylation, another classic mechanism of protein inactivation is a truncating gene mutation, but that does not seem to play a role in prostate carcinoma, as *HNF1B* mutations are a rare event in this type of tumor. In our study, we have found *HNF1B* mutations (VUS – variant of uncertain significance) in 1/77 cases (1.3%) only, which is in accordance with the TCGA study showing only one likely pathogenic mutation in a dataset containing 491 cases of prostate cancer (0.2%).

Only one previous study focused on the assessment of the significance of HNF1B protein expression on immunohistochemical level and its correlation with other variables in prostate carcinoma^51^. This study analyzed 631 samples in tissue microarrays from normal prostate, prostatic intraepithelial neoplasia (PIN), clinically localized cancer (CLC), castration-resistant cancer (CRC), and metastases. They assessed both the cytoplasmic and nuclear staining separately, but the extent of nuclear staining is not further stratified, and the results were only classified as positive or negative. Their results showed that nuclear HNF1B expression is limited in normal prostate (14%), PIN (0%) and CLC (23%), in comparison to the increased HNF1B expression in CRC (96%) and metastases (65%). Their findings were supported by relative mRNA expression, which was low in normal tissue and CLC, and high in CRC. However, our results concerning immunohistochemical findings are very different, with a very low expression of HNF1B detected in both the PC and AH samples (as detected by immunohistochemistry). The explanation for these differences is difficult, and may be related to the methodology of immunohistochemical scoring, because the authors reported the HNF1B expression in a binary manner, with no clear stratification and explanation of their scoring system. Our results are supported by the TCGA, according to which the HNF1B mRNA levels decrease with an increasing Gleason score^11^.

Except for the *HNF1B* promoter methylation, other regulatory mechanisms affecting the *HNF1B* gene expression are not well known. Concerning molecular alterations, prostate carcinoma is a highly heterogeneous disease, in which several driver mutations may occur^11,52^. About 50–70% of prostate carcinomas harbor common chromosomal rearrangements fusing one of the *ETS* transcription family genes (*ERG*, *ETV1*, *ETV4* or *FLI1*) with androgen-regulated genes, most commonly *TMPRSS2*, leading to disruption of androgen receptor signaling via activation of *EZH2*^53^*.* This mechanism, based on repressing the β2 adrenergic receptor pathway, is one of the possible downstream effects of changes of the EZH2 protein levels. Other downstream genes influenced by EZH2 have also been described, including *CDH1* and *DAB2IP*, but the exact downstream genes and pathways have not yet been well established^21,53,54^. Recently, *HNF1B* has been suggested as one of the other possible downstream targets of EZH2^13^*.* In a recent study the authors found that EZH2 binds to the *HNF1B* locus and suppresses the *HNF1B* gene expression in prostate cancer cell lines, with a reverse correlation between EZH2 and HNF1B expression. However, their sample set size is limited to 17 clinical samples and, according to the figure in their manuscript, the HNF1B positive samples showed a rather limited protein expression. Moreover, the authors also found that a higher HNF1B mRNA expression strongly predicted a better prognosis, either alone or combined together with an EZH2 lower expression.

The results of our study confirmed some previously published. In concordance with those, we have found that the expression of EZH2 on an immunohistochemical and mRNA level is statistically significantly higher in prostate carcinoma compared to adenomyomatous hyperplasia samples. An increased expression of EZH2 mRNA showed a statistically significant positive correlation with stage, Gleason score, LVSI, and metastatic disease. An increased expression of EZH2 detected by immunohistochemistry (H-score) showed a statistically significant positive correlation with stage, Gleason score, and BCR. Concerning the relationship between the expression of HNF1B and EZH2 on the mRNA level, the results were not statistically significant in spite of the observed trend of a negative correlation. Nevertheless, this can be related to small sample sets, as the results of a previous study and the re-analysis of TCGA datasets showed a statistically significant negative correlation^11^. On the level of immunohistochemical expression, we have found a significant positive correlation (F = 8.853, p = 0.004) between HNF1B and EZH2 expression, which differs from the results of a previous study which showed a negative correlation in a mouse model and patient sample set. One of the possible explanations for this difference may lie in to the fact that the expression on a protein level and mRNA level may be difficult to correlate for several reasons including translation rates, translation rates modulations, modulations of protein half-life, protein synthesis delay, and protein transport^55^. Moreover, in our recent study, we have shown that there are several transcriptional variants of HNF1B which have not been recognized yet (contrary to the previous knowledge about three possible HNF1B isoforms)^56^. The positive correlation between HNF1B and EZH2 expression in our study was also reflected in the observed positive correlation between AR expression and expression of both EZH2 and HNF1B. Moreover, we have also found significant correlation between the ECI2 and AR expression.Other possible explanation is therefore the formation of an unstable protein isoform, either due to alternative splicing or post-translational modifications. Due to this, the correlation on a protein level does not necessarily reflect the relationship with mRNA level (which was measured as the sum of all HNF1B mRNA variants, due to the target in 3′ UTR of HNF1B mRNA). Given that, we believe that the protein level expression, as the final step of gene expression, reflects the relationship between HNF1B and EZH2 gene expression more accurately, regardless of our and previously described mRNA expression results, which may be limited due to the aforementioned reasons. Our findings should be supported by a larger data set analysis, in order to exclude the possibility that the result showing the positive correlation of HNF1B and EZH2 expression on a protein level may have been influenced by the relatively small sample set.

The downstream interaction of HNF1B is currently poorly understood, but based on the animal model it has been suggested that the downregulation of HNF1B during tumor progression is associated with the upregulation of enoyl-CoA-(Δ) isomerase 2 (ECI2) protein levels, which is one of the possible downstream targets of HNF1B^31^. *ECI2* represents one of the androgen receptor (AR) target genes^32,57^. The results of a recent study showed that ECI2 is overexpressed in prostate carcinoma on both mRNA and protein levels (detected by immunohistochemistry), and that the increased gene expression of *ECI2* predicts statistically significant poor outcome (survival), as analyzed on 144 patient samples^32^. According to this study, which also focused on cell cultures, the inhibition of *ECI2* gene expression led to acute metabolic stress in prostate cancer cells in the cell culture, and the targeting of ECI2 (or more generally lipid degradation) may have a therapeutic significance, although the exact mechanism(s) by which *ECI2* knockdown inhibits cancer cell proliferation is unknown.

In a mouse model, the authors compare HNF1B and ECI2 protein expression at different stages of prostate cancer development and concluded that during tumor progression, the protective effect of HNF1B is lost, and that is associated with an increased expression of ECI2^31^. The results of our study, compared to the only two previous studies concerning ECI2 in prostate cancer, similarly found an overexpression of ECI2 in the PC group, which was statistically significant in contrast to the low expression in AH samples. Contrary to the one previously published study, the results of which showed that increased ECI2 expression is a poor prognostic marker for overall survival, we did not confirm the prognostic meaning of ECI2 for any of the analyzed outcomes. We also did not find any statistically significant correlation between HNF1B and ECI2 expressions on a protein level, in contrast to the results described in one study performed on an animal model^31^. However, we have found a positive correlation between ECI2 expression and EZH2 expression, as detected by immunohistochemistry. Concerning mRNA expression, we have found a negative correlation trend between the HNF1B and EZH2 mRNA expression, and also between the HNF1B and ECI2 mRNA expression which, however, was not statistically significant in our dataset. We have also detected a significant, positive correlation between the ECI2 and EZH2 mRNA expression.

In conclusion, the results of our study showed that the expression of HNF1B on an IHC level is very low in prostate carcinoma, does not differ between PC and AH, and did not correlate with any clinical outcomes. We have found that the methylation of the *HNF1B* promoter, in concordance with previous studies, is a common finding in PC and showed a positive correlation with Gleason score and stage. Compared to PC, the methylation detected in AH was very low in our study (6%). We have found that the expression of EZH2 (based on both IHC and mRNA levels) was higher in PC compared to AH and, in concordance with previous studies, it is a negative prognostic sign which was correlated with most of the clinical outcomes. Our study is also the first instance when the relationship between EZH2 and ECI2 was analyzed, and our results showed that they are positively correlated on both mRNA expression and protein expression levels. Contrary to EZH2, ECI2 did not correlate with any of the clinical outcomes. The other upstream and downstream regulatory mechanisms in HNF1B pathway(s) may encompass EZH2 and ECI2, as suggested by two previous studies, but the relationship between HNF1B and EZH2/ECI2 in our in vivo study focusing on human samples was ambiguous. We have found a negative correlation trend between HNF1B and EZH2/ECI2 mRNA expression, which was, however, not statistically significant. Surprisingly, we have also found a positive correlation between HNF1B and EZH2 expression on an immunohistochemical level, which was not correlated with the mRNA levels. However, those results were in concordance with immunohistochemical expression of AR, which showed positive significant correlation with both EZH2 and HNF1B. Nevertheless, this finding requires further analysis on a larger sample set. Our results support the oncosuppressive role of HNF1B in prostate carcinoma, which may be silenced due to promoter methylation and other mechanisms, but not due to gene mutation, which seems to be very rare in PC. Despite the equivocal relations between HNF1B, EZH2 and ECI2, the high expression of EZH2 and ECI2 in PC seems to be a potential therapeutic target, especially in the case of EZH2 as suggested in previous studies^17,58–63^.

## Material and methods

### Samples

For the purposes of the study we primarily used formalin-fixed paraffin-embedded (FFPE) tissue blocks and, where available, the corresponding fresh-frozen tissue (FT) for the molecular DNA/RNA analysis. The FFPE tissue blocks were obtained from the archives of our department, and the corresponding FT samples were provided by the Bank of Biological Material (BBM) of the First Faculty of Medicine, Charles University in Prague. The FT tissue samples were macro-dissected from surgically resected tissue samples by a trained pathologist, stabilized immediately in the RNAlater stabilization solution (Qiagen), and stored at − 80 °C according to the manufacturer´s protocol (Stabilization of RNA in Harvested Animal Tissues; Qiagen) as described in our previous study^64^. A total of 119 FFPE tissue samples were used for the immunohistochemical analysis, including 101 cases of PC, and 18 cases of AH. From the total of 119 FFPE cases, there were 73 cases with available corresponding FT samples, including all 18 AH samples and 55 PC samples (from which paired non-tumor tissue was available in 49 cases).

The clinico-pathological characteristics of the analyzed PC samples are summarized in Table 1.

The PC samples were divided according to the TNM classification into four groups (T1-T4), and according to the Gleason score into three grade groups: low grade (grade group 1), intermediate grade (grade group 2 and 3), and high grade (grade group 4 and 5).

All the included cases underwent a histologic review of the hematoxylin and eosin-stained slides. During this review, the eligible and appropriate areas of the tumor were identified and marked in order to provide tissue cores for the construction of the TMAs. Two tissue cores (each 2.0 mm in diameter) were drilled from the donor block from each case using the tissue microarray instrument TMA Master (3DHISTECH Ltd., Budapest, Hungary).

### DNA and RNA isolation, quality control and cDNA synthesis

Prior to the isolation of the FT samples, the tissues were thawed, and 10–30 mg were homogenized using MagNA Lyser Green Beads tubes in a MagNA Lyser Instrument (Roche) in the presence of 600 µl of RLT Plus buffer (Qiagen) with 6 µl of 14.3 M 2-mercaptoethanol (Sigma-Aldrich). The total DNAs and RNAs were isolated according to the Simultaneous Purification of Genomic DNA and Total RNA from Animal Tissues protocol by using an AllPrep DNA/RNA Mini kit (Qiagen). The isolated DNA and RNA samples were quantified by NanoDrop 2000 (Thermo Fisher).

The total RNA was subjected to further RNA quality analysis using Fragment Analyzer (AATI) capillary electrophoresis system and Standard RNA kit (AATI), resulting in RNA Quality Number (RQN; tissue samples RQN mean = 9.6; range 7.1–10). All RNA samples were subjected to further analysis.

After RNA quality characterization, 3.75 µg of the total RNA of each sample (where available) was treated by DNase I (Thermo Fisher) prior to one-step cDNA synthesis in 40 µl reaction using SuperScript III Reverse Transcriptase (Thermo Fisher) with random hexamers (Roche) as described previously^56^. All RNA samples were processed according to the Digital MIQE Guidelines^65^.

DNA from 5–10 tissue sections (5 µm) from the archived FFPE tissue blocks was isolated using an automatic isolator MagCore Nucleic Acid Extractor, utilizing the MagCore Genomic DNA FFPE One-step kit, Ref MGF-03 (RBC Bioscience). The isolated DNA was quantified by Qubit fluorimeter (Thermo Fisher) and underwent a quality control test of amplification efficacy by qPCR (5 ng of the DNA sample was amplified using 5 × HOT FIREPol EvaGreen HRM Mix NO ROX; Solis Biodyne) as we described elsewhere ^66^. Only the samples which passed the quality criteria (Cp < 35 for a 180 bp product amplification; 24/46 samples) were used for the subsequent analysis.

### Immunohistochemical analysis

The immunohistochemical (IHC) analysis was performed on all samples using the standard 4 μm thick sections of FFPE tissue and the automated staining instrument Ventana BenchMark ULTRA (Roche, Basel, Switzerland) with the following rabbit antibodies: HNF1B (polyclonal, dilution 1:500, Sigma-Aldrich, Prestige Antibodies, St. Louis, United States); EZH2 (clone D2C9, dilution 1:200, Cell Signalling, Danvers, MA, USA), ECI2 (polyclonal, dilution 1:100, Abcam, Cambridge, United Kingdom), and androgen receptor (monoclonal, clone AB1, dilution 1:100, Invitrogen, Carlsbad, CA, USA). The heat induced epitope retrieval with a citrate buffer (pH 6.0) was used for pre-treatment. The detection of the primary antibody was visualized using the OptiView (HNF1B) and UltraView (EZH2 and ECI2) Universal DAB Detection Kit (Ventana, Roche). Only nuclear staining was regarded as positive for HNF1B and EZH2. For ECI2, the cytoplasmic staining was evaluated.

The immunohistochemical results were assessed according to the overall percentage of positive cells (0–100%) and then also semi-quantitatively, using the H-score as described in our previous study^64^. This method is based on the assessment of the percentage of positive cells based on the level of staining intensity (1 + for weak intensity, 2 + for moderate and 3 + for strong intensity). The final H-score for each case is then calculated by adding the multiplication of the different staining intensities according to the following formula: [1 × (% of cells 1 +) + 2 × (% of cells 2 +) + 3 × (% of cells 3 +)], resulting in the H-score value of 0–300.

### Molecular analysis of DNA

DNA analysis included mutation analysis of the coding parts of the *HNF1B* exons with adjacent intronic sequences (± 15 bp) and intronic regions containing the rs7527210 and rs4430796 polymorphisms and epigenetic analysis of CpG methylation in the region of the *HNF1B* promoter.

The *HNF1B* mutation analysis was performed using two different next-generation sequencing (NGS) approaches, depending on the type of tissue available. For 53 tumor samples and 49 corresponding non-tumor tissue for which the FT was available, the DNA samples were amplified by in-house 2-step polymerase chain reaction (PCR), and analyzed by amplicon next-generation sequencing (NGS). The 24 samples with only FFPE tissue available, which passed the DNA quality control criteria, were analyzed by a capture-based panel NGS, which is more suitable for FFPE samples and included all the coding parts of the *HNF1B* gene.

### Amplicon NGS preparation and sequencing

For the in-house 2-step PCR amplicon approach, 15 primer pairs with universal adaptor sequences were designed (the list of primers is provided in Supplementary Table 3) to fit the specific *HNF1B* gene regions in the first PCR step, including deep intronic regions containing the rs7527210 and rs4430796 variants. In the second PCR step, a universal primer pair containing Illumina sequencing adaptor sequences was used. The first PCR step covering the *HNF1B* target regions was performed in two separate multiplex reactions. Each reaction included a different primer pair set in order to eliminate unwanted primer interactions. Both PCR reactions were amplified using the FastStart High Fidelity PCR System (Roche) according to the recommended standard PCR procedure (FastStart High Fidelity PCR System; Roche) in 20 µl reactions according to the following PCR protocol: 2 min—95 °C; 10 cycles of 15 s—95 °C, 20 s—62 °C and 30 s—72 °C (all steps with ramping temperature 2 °C/s) and then 20 cycles of 15 s—95 °C, 20 s—62 °C and 30 s—72 °C (standard ramping temperature 4 °C/s). After the first PCR step, 10 µl of both first PCR reactions were equimolarly mixed and purified by the AMPure XP system (0.8 ×; Beckmann Coulter). The purified PCR product was amplified by 10 cycles of the second PCR step using the same protocol, with a standard ramping rate and different primers (primer pairs containing universal Illumina adaptor and molecular barcode sequences unique for each sample).

After the second PCR step, the concentrations of the PCR products were measured using the Qubit fluorimeter (Thermo Fisher) and then equimolarly mixed into one sequencing library. Once the sequencing library was prepared, it was purified using the AMPure XP system (0.8x; Beckmann Coulter), measured for concentration (Qubit) and for fragment length using High Sensitivity NGS Fragment Analysis kit on the Fragment Analyzer (AATI). The amplicon library was then sequenced together with different (capture) libraries in order to increase sequencing heterogeneity. The sequencing was performed either using 50 samples, which were sequenced in one amplicon library by the MiSeq 300 cycles v2 kit or using 90 samples by the NextSeq 300 cycles mid output kit v2.5. The amplicon sequencing approach showed low coverage of 20 bp on the 5′ end of the exon 4 in all of the tested samples, and therefore this part was additionally sequenced by the Sanger sequencing method as we described elsewhere with the use of a specific primer pair (Supplementary Table 3)^67^.

### Capture-based NGS preparation and sequencing

The DNA from the FFPE samples was prepared using the SeqCap custom hybridization probes (257kbp panel of gene targets, NimbleGen, Roche), and sequenced as we described elsewhere^66^. The libraries were sequenced by the NextSeq 500 instrument (Illumina, San Diego, CA, USA) using the NextSeq 500/550 Mid Output Kit v2.5 (150 Cycles; Illumina).

### Biostatistical analysis of NGS data

Both the amplicon and panel sequencing raw data were demultiplexed and converted into the .fastq format and analyzed by the same pipeline using the NextGENe software (Softgenetics) as described elsewhere^66^. For the reads mapping and analysis, the GRCh37 genome and NM_000458.2 reference transcript was used. Only the samples with minimal coverage > 200 × and variants with variant allele frequency (VAF) > 10% were further evaluated. The identified variants were manually inspected using IGV (Broad Institute) and prioritized according to the mutation impact^66^. Only the mutations of class 3, 4 or 5 were reported.

### The *HNF1B* promoter methylation analysis

The bisulfite conversion of DNA was performed using the EZ DNA Methylation-Lightning Kit (Zymo Research, Irvine, CA, USA) according to the manufacturer’s instructions. The primers (Supplementary Table 3) used for the PCR amplification of both the methylated and unmethylated alleles were designed using the software Methprimer (https://www.urogene.org/cgi-bin/methprimer/methprimer.cgi). The amplified promoter region of *HNF1B* covers 15 CpG islands (the PCR product corresponding to the relative transcription start site -457 to -202, GRCh37), including a CpG island (chr17:36105517–36105518, GRCh37). In our setting, we were able to detect at least 5% of methylated DNA by High Resolution Melting (HRM) Analysis of the amplified PCR products. Each run included the converted DNA samples and a series of 100%, 20%, 10%, 5 and 0% universally methylated DNA controls mixed with non-methylated DNA (Human HCT116 DKO Non-Methylated DNA and Human HCT116 DKO Methylated DNA; Zymo Research). The melting curves of the analyzed samples were compared with the melting curves of the control mixes, as described elsewhere^68^. Due to the unspecified quantity of tumor tissue in the sample if the methylation was detected by HRM analysis, the sample was considered to be methylated.

### Expression mRNA analysis

The expression analysis of cDNA (synthesized from the total RNA as described above) was performed using the droplet digital PCR (ddPCR) system (Bio-Rad). Prior to the general ddPCR analysis, several optimization steps including the testing of expression of three pre-selected reference gene mRNA targets (POLR2A, HPRT1 and ATP5F1B), two HNF1B mRNA targets (in 5′ UTR and 3′ UTR), repeatability, reproducibility and optimal primers annealing temperature, were performed.

Droplet digital PCR reactions were prepared using QX200 ddPCR EvaGreen Supermix (Bio-Rad; according to the manufacturer’s instructions), 1 µl of cDNA template (which corresponds to approx. 90 ng of the total RNA) and 4 pmol of each of the primer pairs (200 nM final concentration) in 20 µl reaction volume. After master mix droplet generation in the QX200 AutoDG instrument (BioRad), the samples were amplified according to the manufacturer’s protocol (5 min incubation at 95 °C followed by 40 cycles of 95 °C for 30 s and 58 °C for 1 min, and final signal stabilization steps consisting of 4 °C for 5 min followed by 90 °C for 5 min). The resulting data was acquired using QX200 Droplet Reader instrument (BioRad) by the standard acquisition protocol for EvaGreen master mix, and analyzed by the QuantaSoft software (BioRad). The threshold for positive droplet signals of each of the four final amplicons (reference POLR2A; HNF1B 3′ UTR, EZH2 and ECI2 targets) was set as the average of the thresholds which were calculated automatically by QuantaSoft software during the optimization steps. The thresholds of all the acquired targets were manually confirmed. The final data of targets (HNF1B, EZH2 and ECI2), expressed as number of templates in 20 µl of master mix (which corresponds to 1 µl of cDNA), were re-calculated as the number of targets per one thousand of the reference POLR2A target, and analyzed as described in the Statistics section.

### Statistical analyses

Statistical analyses were performed using the software Statistica (TIBCO). The Shapiro–Wilk test was used to control data normality. The nonparametric ANOVA approach was used in order to analyze the association between HNF1B expression (H-score as a continuous dependent variable) and clinico-pathological characteristics (categorical variables). Depending on the number of categories either the Mann–Whitney U-test (two categories) or the Kruskal–Wallis H-test (three and more categories) was used. For the evaluation of the effect of independent clinico-pathological characteristics on the categorized H-score or methylation status, the Pearson chi-square test was used. Correlations between two continuous variables were analyzed using Pearson’s method. The evaluated clinico-pathological variables included age, stage, Gleason score, resection margin, LVSI, perineural invasion, metastases, preoperative PSA level, biochemical recurrence, methylation status and rs4430796.

BCR was defined as serum prostate-specific antigen (PSA) ≥ 0.2 ng/ml after radical prostatectomy. The time of BCR was defined as the earliest time after surgery at which the postoperative serum PSA achieved this concentration. The prognostic factors of BCR were calculated using uni- and multivariable analyses in a Kaplan–Meier model and Cox proportional hazards model. The survival curve differences were evaluated using log-rank test.

For the purposes of chi-squared tests and survival analyses, the H-score was categorized into two groups: (i) HNF1B (range = 0–160, mean = 19, median = 0): group 1 = H-score ˂ 10, group 2 = H-score ≥ 10; (ii) EZH2 (range = 0–155, mean = 40, median = 30): group 1 = H-score ˂ 30, group 2 = H-score ≥ 30; (iii) ECI2 (range = 0–300, mean = 152, median = 150): group 1: H-score ˂ 150, group 2 = H-score ≥ 150).

All tests were two-sided and a p-value of less than 0.05 was considered as significant.

### Analysis of the prostate carcinoma samples of The Cancer Genome Atlas (TCGA) dataset

The data of TCGA including the clinico-pathological findings and mRNA expression (z-score) of HNF1B, EZH2 and ECI2 was downloaded through cBioPortal (www.cbioportal.org; Prostate Adenocarcinoma (TCGA, Firehose Legacy, access March 2020)). Additionally, the data on promoter methylation of these genes was downloaded through the Mexpress (https://mexpress.be). The methylation (beta value) levels of the CpG islands of the promoter regions which most negatively correlated with mRNA expression of the relevant gene (i.e. cg12788467 for *HNF1B*, cg02303805 for *EZH2* and cg12928997 for *ECI2*) were chosen for the subsequent statistical analysis.

### Ethical approval

The study has been approved by the Ethics Committee of General University Hospital in Prague in compliance with the Helsinki Declaration (ethical approval number 41/16 Grant VES 2017 AZV VFN). The Ethics Committee waived the requirement for informed consent, because according to the Czech Law (Act. no. 373/11, and its amendment Act no. 202/17) it is not necessary to obtain informed consent in fully anonymized studies.

## Supplementary information

Supplementary Table 1.

Supplementary Table 2.

Supplementary Table 3.

## Data Availability

All data generated or analyzed during this study are included in this published article (and its Supplementary Information files).
