# Supplementary material for: HNF1B, EZH2 and ECI2 in prostate carcinoma. Molecular, immunohistochemical and clinico-pathological study
Source: Sci Rep. 2020 Sep 1;10:14365. doi: 10.1038/s41598-020-71427-7 (PMC7463257; doi:10.1038/s41598-020-71427-7)
Supplement: Supplementary file 1 — Supplementary Table 1. [file 41598_2020_71427_MOESM1_ESM.docx]

HNF1B, EZH2 and ECI2 in prostate carcinoma. Molecular, immunohistochemical and clinico-pathological study.

Running title: HNF1B, EZH2 and ECI2 in prostate carcinoma

Pavel Dundr^1*^, Michaela Bártů^1^, Jan Hojný^1^, Romana Michálková^1^, Nikola Hájková^1^, Ivana Stružinská^1^, Eva Krkavcová^1^, Ladislav Hadravský^2^, Lenka Kleissnerová^1^, Jana Kopejsková^1^, Bui Quang Hiep^1^, Kristýna Němejcová^1^, Radek Jakša^1^, Otakar Čapoun^3^, Jakub Řezáč^3^, Kateřina Jirsová^4^, Věra Franková^5^

^1^Institute of Pathology, First Faculty of Medicine, Charles University and General University Hospital in Prague, Czech Republic

^2^Institute of Pathology, First Faculty of Medicine, Charles University, Czech Republic

^3^Department of Urology, First Faculty of Medicine, Charles University and General University Hospital in Prague, Czech Republic

^4^Institute of Biology and Medical Genetics, First Faculty of Medicine, Charles University and General University Hospital in Prague, Czech Republic

^5^Department of Pediatrics and Adolescent Medicine, First Faculty of Medicine, Charles University and General University Hospital in Prague, Czech Republic

*Corresponding author:

Pavel Dundr, M.D., Ph.D.

Institute of Pathology, First Faculty of Medicine, Charles University and General University Hospital in Prague, Studničkova 2, 12800 Prague 2, Czech Republic

Email: [pavel.dundr@vfn.cz](mailto:pavel.dundr@vfn.cz)

Supplementary table 1. Raw data (immunohistochemistry and mRNA expression)

| Case | Daignosis | IHC _HNF1B % | IHC _HNF1B 1+ | IHC _HNF1B 2+ | IHC_HNF1B 3+ | IHC_EZH2 % | IHC_EZH2 1+ | IHC_EZH2 2+ | IHC_EZH2 3+ | IHC_ECI2 % | IHC_ECI2 1+ | IHC_ECI2 2+ | IHC_ECI2 3+ | ddPCR2_tumor POLR2A | ddPCR2_tumor EZH2 | ddPCR2_tumor  HNF1B | ddPCR3_tumor POLR2A | ddPCR3_tumor ECI2 | ddPCR3_tumor HNF1B |
| --- | --- | --- | --- | --- | --- | --- | --- | --- | --- | --- | --- | --- | --- | --- | --- | --- | --- | --- | --- |
| 1 | PC | 0 | 0 | 0 | 0 | 5 | 5 | 0 | 0 | 100 | 70 | 30 | 0 | 786 | 23,7 | 16,8 | 648 | 131,5 | 11,9 |
| 2 | PC | 0 | 0 | 0 | 0 | 0 | 0 | 0 | 0 | 100 | 30 | 70 | 0 | NULL | NULL | NULL | NULL | NULL | NULL |
| 3 | PC | 0 | 0 | 0 | 0 | 40 | 10 | 20 | 10 | 100 | 60 | 40 | 0 | 1688 | 258 | 10,7 | 1760 | 227 | 7,7 |
| 4 | PC | 80 | 70 | 10 | 0 | 15 | 5 | 5 | 5 | 100 | 20 | 80 | 0 | 1316 | 59,3 | 49,1 | 1284 | 339 | 33,5 |
| 5 | PC | 90 | 90 | 0 | 0 | 15 | 10 | 5 | 0 | 100 | 0 | 100 | 0 | 1885 | 29,8 | 97,8 | 1755 | 173 | 74,6 |
| 6 | PC | 0 | 0 | 0 | 0 | 10 | 5 | 5 | 0 | 100 | 10 | 90 | 0 | 1138 | 47,8 | 58,8 | 1197 | 272 | 50,7 |
| 7 | PC | 0 | 0 | 0 | 0 | 7 | 5 | 2 | 0 | 100 | 0 | 70 | 30 | 1446 | 24,5 | 68 | 1387 | 115,7 | 58,8 |
| 8 | PC | 0 | 0 | 0 | 0 | 5 | 5 | 0 | 0 | 100 | 100 | 0 | 0 | 817 | 172,2 | 37,9 | 861 | 341 | 32 |
| 9 | PC | 0 | 0 | 0 | 0 | 10 | 5 | 5 | 0 | 100 | 60 | 40 | 0 | 720 | 36,5 | 44,9 | 658 | 116,4 | 33,5 |
| 10 | PC | 0 | 0 | 0 | 0 | 70 | 50 | 20 | 0 | 100 | 10 | 50 | 40 | 522 | 30 | 6 | 577 | 236 | 3,8 |
| 11 | PC | 0 | 0 | 0 | 0 | 15 | 10 | 5 | 0 | 100 | 20 | 80 | 0 | 754 | 57,6 | 92 | 665 | 207 | 75,4 |
| 12 | PC | 0 | 0 | 0 | 0 | 2 | 2 | 0 | 0 | 100 | 20 | 80 | 0 | 960 | 26,9 | 56,5 | 1035 | 114,3 | 51,5 |
| 13 | PC | 30 | 30 | 0 | 0 | 40 | 30 | 5 | 5 | 100 | 0 | 90 | 10 | 1200 | 67,3 | 75,8 | 1190 | 129,1 | 66,4 |
| 14 | PC | 0 | 0 | 0 | 0 | 25 | 20 | 5 | 0 | NA | 0 | 0 | 0 | 1526 | 49,4 | 63,3 | 1478 | 377 | 47,1 |
| 15 | PC | 10 | 10 | 0 | 0 | 50 | 35 | 15 | 0 | 100 | 50 | 50 | 0 | 987 | 21,4 | 65,3 | 1302 | 123,6 | 64,6 |
| 16 | PC | 0 | 0 | 0 | 0 | 45 | 30 | 10 | 5 | 100 | 0 | 20 | 80 | 1493 | 50,3 | 5,5 | 1507 | 539 | 6 |
| 17 | PC | 0 | 0 | 0 | 0 | 60 | 30 | 20 | 10 | 100 | 0 | 60 | 40 | 847 | 382 | 7,2 | 701 | 241 | 5,6 |
| 18 | PC | 0 | 0 | 0 | 0 | 90 | 40 | 40 | 10 | 100 | 0 | 20 | 80 | 1030 | 143,8 | 4,8 | 1078 | 207 | 4,6 |
| 19 | PC | 0 | 0 | 0 | 0 | 0 | 0 | 0 | 0 | 20 | 20 | 0 | 0 | 1221 | 18,6 | 70,3 | 1023 | 139,8 | 52,4 |
| 20 | PC | 0 | 0 | 0 | 0 | 15 | 10 | 5 | 0 | 100 | 0 | 100 | 0 | 998 | 25,4 | 41 | 1060 | 134,2 | 35,2 |
| 21 | PC | 0 | 0 | 0 | 0 | 5 | 5 | 0 | 0 | 100 | 0 | 80 | 20 | 933 | 27,3 | 39 | 924 | 138,8 | 33,5 |
| 22 | PC | 0 | 0 | 0 | 0 | 0 | 0 | 0 | 0 | 100 | 90 | 10 | 0 | 636 | 15,5 | 25,4 | 542 | 89,4 | 20,6 |
| 23 | PC | 90 | 90 | 0 | 0 | 35 | 20 | 10 | 5 | 100 | 10 | 60 | 30 | 870 | 52 | 116,5 | 731 | 139,6 | 89,5 |
| 24 | PC | 0 | 0 | 0 | 0 | 15 | 10 | 5 | 0 | 100 | 0 | 100 | 0 | 1363 | 17,4 | 71,8 | 1223 | 143,9 | 54,6 |
| 25 | PC | 0 | 0 | 0 | 0 | 0 | 0 | 0 | 0 | 20 | 20 | 0 | 0 | 1546 | 27 | 84,8 | 1377 | 99,6 | 61,6 |
| 26 | PC | 0 | 0 | 0 | 0 | 25 | 20 | 5 | 0 | 100 | 0 | 100 | 0 | 773 | 45,2 | 145,1 | 633 | 158 | 122 |
| 27 | PC | 70 | 70 | 0 | 0 | 60 | 30 | 25 | 5 | 100 | 0 | 100 | 0 | 861 | 65,8 | 201 | 776 | 296 | 181 |
| 28 | PC | 0 | 0 | 0 | 0 | 3 | 3 | 0 | 0 | 70 | 70 | 0 | 0 | 1483 | 56,1 | 128,8 | 1464 | 348 | 104,5 |
| 29 | PC | 0 | 0 | 0 | 0 | 10 | 10 | 0 | 0 | 100 | 60 | 40 | 0 | 995 | 45,2 | 46 | 982 | 136,7 | 41,8 |
| 30 | PC | 0 | 0 | 0 | 0 | 25 | 20 | 5 | 0 | 100 | 30 | 70 | 0 | 1435 | 22,4 | 63,7 | 1391 | 241 | 58,3 |
| 31 | PC | 90 | 80 | 10 | 0 | 15 | 10 | 5 | 0 | 100 | 80 | 20 | 0 | 1517 | 24,1 | 74,2 | 1174 | 113,5 | 51,9 |
| 32 | PC | 90 | 90 | 0 | 0 | 20 | 10 | 10 | 0 | 100 | 20 | 60 | 20 | 1356 | 15 | 71,7 | 1526 | 68,2 | 68,5 |
| 33 | PC | 0 | 0 | 0 | 0 | 3 | 0 | 0 | 0 | 100 | 70 | 30 | 0 | 1297 | 28,9 | 83,4 | 1373 | 155,4 | 66,1 |
| 34 | PC | 0 | 0 | 0 | 0 | 55 | 30 | 20 | 5 | 100 | 0 | 100 | 0 | 1468 | 94,3 | 87,8 | 1441 | 353 | 72,1 |
| 35 | PC | 0 | 0 | 0 | 0 | 33 | 20 | 10 | 3 | 100 | 0 | 100 | 0 | 1240 | 20,5 | 58,2 | 1044 | 118 | 46,2 |
| 36 | PC | 30 | 30 | 0 | 0 | 15 | 10 | 5 | 0 | 100 | 80 | 20 | 0 | 1005 | 41,7 | 53,4 | 922 | 198 | 47,7 |
| 37 | PC | 90 | 40 | 50 | 0 | 60 | 50 | 5 | 5 | 100 | 60 | 40 | 0 | 1422 | 30,9 | 58,9 | 1476 | 173 | 47,4 |
| 38 | PC | 0 | 0 | 0 | 0 | 10 | 10 | 0 | 0 | 100 | 20 | 80 | 0 | 1782 | 39,2 | 42 | 1672 | 329 | 36,2 |
| 39 | PC | 0 | 0 | 0 | 0 | 35 | 20 | 10 | 5 | 100 | 100 | 0 | 0 | 853 | 36,8 | 36,4 | 771 | 144 | 29,6 |
| 40 | PC | 70 | 40 | 30 | 0 | 40 | 30 | 5 | 5 | 100 | 70 | 30 | 0 | 1449 | 58,4 | 162 | 1374 | 267 | 142 |
| 41 | PC | 0 | 0 | 0 | 0 | 10 | 5 | 5 | 0 | 100 | 0 | 100 | 0 | 1437 | 26 | 90,2 | 1362 | 220 | 77,9 |
| 42 | PC | 5 | 5 | 0 | 0 | 40 | 25 | 10 | 5 | 100 | 50 | 50 | 0 | 1280 | 52,9 | 73 | 1171 | 399 | 65,4 |
| 43 | PC | 30 | 30 | 0 | 0 | 80 | 60 | 10 | 10 | 100 | 0 | 100 | 0 | 1232 | 45,1 | 61,7 | 1079 | 304 | 42 |
| 44 | PC | 0 | 0 | 0 | 0 | 80 | 40 | 30 | 10 | 100 | 100 | 0 | 0 | 1222 | 331 | 29,9 | 1183 | 330 | 20,6 |
| 45 | PC | 0 | 0 | 0 | 0 | 15 | 10 | 5 | 0 | 100 | 80 | 20 | 0 | 1067 | 59,1 | 40,8 | 1083 | 171 | 30,4 |
| 46 | PC | 0 | 0 | 0 | 0 | 75 | 50 | 20 | 5 | 100 | 80 | 20 | 0 | 68,4 | 1,03 | 0,3 | 74,5 | 24,7 | 0,91 |
| 47 | PC | 0 | 0 | 0 | 0 | 65 | 50 | 10 | 5 | 100 | 70 | 30 | 0 | 1338 | 185 | 324 | 1484 | 355 | 107 |
| 48 | PC | 90 | 20 | 70 | 0 | 90 | 50 | 30 | 10 | 100 | 60 | 40 | 0 | 1745 | 22 | 70,4 | 1968 | 272 | 61,6 |
| 49 | PC | 0 | 0 | 0 | 0 | 70 | 30 | 20 | 20 | 100 | 80 | 20 | 0 | 723 | 13,6 | 33,8 | 739 | 80,9 | 35,1 |
| 50 | PC | 0 | 0 | 0 | 0 | 15 | 10 | 5 | 0 | 100 | 80 | 20 | 0 | 1185 | 47,5 | 52,3 | 1304 | 376 | 48,8 |
| 51 | PC | 0 | 0 | 0 | 0 | 15 | 10 | 5 | 0 | 100 | 0 | 20 | 80 | 733 | 35,3 | 50,9 | 656 | 150 | 45,2 |
| 52 | PC | 0 | 0 | 0 | 0 | 3 | 3 | 0 | 0 | 100 | 20 | 60 | 20 | 1059 | 29 | 45,3 | 1146 | 489 | 40,7 |
| 53 | PC | 0 | 0 | 0 | 0 | 30 | 20 | 10 | 0 | 100 | 0 | 100 | 0 | 1123 | 35,2 | 65,6 | 1088 | 268 | 68,5 |
| 54 | PC | 0 | 0 | 0 | 0 | 30 | 20 | 10 | 0 | 100 | 80 | 20 | 0 | 982 | 30,6 | 92,2 | 995 | 259 | 77,2 |
| 55 | PC | 0 | 0 | 0 | 0 | 0 | 0 | 0 | 0 | 100 | 0 | 100 | 0 | 328 | 25,7 | 0 | 421 | 353 | 1,11 |
| 56 | PC | 0 | 0 | 0 | 0 | 0 | 0 | 0 | 0 | 100 | 20 | 80 | 0 | NULL | NULL | NULL | NULL | NULL | NULL |
| 57 | PC | 0 | 0 | 0 | 0 | 0 | 0 | 0 | 0 | 50 | 50 | 0 | 0 | NULL | NULL | NULL | NULL | NULL | NULL |
| 58 | PC | 0 | 0 | 0 | 0 | 5 | 5 | 0 | 0 | 100 | 40 | 60 | 0 | NULL | NULL | NULL | NULL | NULL | NULL |
| 59 | PC | 10 | 10 | 0 | 0 | 20 | 15 | 5 | 0 | 100 | 20 | 80 | 0 | NULL | NULL | NULL | NULL | NULL | NULL |
| 60 | PC | 0 | 0 | 0 | 0 | 3 | 2 | 1 | 0 | 100 | 80 | 20 | 0 | NULL | NULL | NULL | NULL | NULL | NULL |
| 61 | PC | 0 | 0 | 0 | 0 | 5 | 3 | 2 | 0 | 100 | 50 | 50 | 0 | NULL | NULL | NULL | NULL | NULL | NULL |
| 62 | PC | 0 | 0 | 0 | 0 | 35 | 20 | 10 | 5 | 100 | 80 | 20 | 0 | NULL | NULL | NULL | NULL | NULL | NULL |
| 63 | PC | 0 | 0 | 0 | 0 | 20 | 15 | 5 | 0 | 100 | 30 | 70 | 0 | NULL | NULL | NULL | NULL | NULL | NULL |
| 64 | PC | 0 | 0 | 0 | 0 | 10 | 10 | 0 | 0 | 100 | 20 | 70 | 10 | NULL | NULL | NULL | NULL | NULL | NULL |
| 65 | PC | 0 | 0 | 0 | 0 | 7 | 5 | 2 | 0 | 100 | 100 | 0 | 0 | NULL | NULL | NULL | NULL | NULL | NULL |
| 66 | PC | 0 | 0 | 0 | 0 | 40 | 25 | 15 | 0 | 100 | 50 | 30 | 20 | NULL | NULL | NULL | NULL | NULL | NULL |
| 67 | PC | 0 | 0 | 0 | 0 | 0 | 0 | 0 | 0 | 70 | 70 | 0 | 0 | NULL | NULL | NULL | NULL | NULL | NULL |
| 68 | PC | 0 | 0 | 0 | 0 | 5 | 5 | 0 | 0 | 100 | 50 | 50 | 0 | NULL | NULL | NULL | NULL | NULL | NULL |
| 69 | PC | 0 | 0 | 0 | 0 | 55 | 40 | 10 | 5 | 100 | 30 | 70 | 0 | NULL | NULL | NULL | NULL | NULL | NULL |
| 70 | AH | 0 | 0 | 0 | 0 | 6 | 5 | 1 | 0 | 70 | 70 | 0 | 0 | 1120 | 12,4 | 59,6 | 1232 | 114 | 55,7 |
| 71 | AH | 0 | 0 | 0 | 0 | 5 | 5 | 0 | 0 | 80 | 80 | 0 | 0 | 1140 | 14,2 | 49,6 | 1110 | 121 | 48,8 |
| 72 | AH | 0 | 0 | 0 | 0 | 3 | 2 | 1 | 0 | 80 | 70 | 10 | 0 | 5,4 | 0,07 | 0,08 | 7,1 | 1,9 | 0,12 |
| 73 | AH | 20 | 20 | 0 | 0 | 6 | 3 | 3 | 0 | 100 | 90 | 10 | 0 | 99,5 | 2,6 | 0,16 | 91,3 | 29 | 0,35 |
| 74 | AH | 30 | 20 | 10 | 0 | 2 | 1 | 1 | 0 | 100 | 100 | 0 | 0 | 141 | 3,2 | 3,7 | 118 | 24,7 | 3,1 |
| 75 | AH | 20 | 20 | 0 | 0 | 0 | 0 | 0 | 0 | 90 | 90 | 0 | 0 | 70,1 | 0,5 | 1,6 | 41,5 | 11,7 | 0,94 |
| 76 | AH | 30 | 20 | 10 | 0 | 2 | 0 | 0 | 0 | 100 | 100 | 0 | 0 | 1710 | 19,8 | 72,1 | 1502 | 129 | 65,1 |
| 77 | AH | 0 | 0 | 0 | 0 | 0 | 0 | 0 | 0 | 100 | 100 | 0 | 0 | 1154 | 15 | 55,5 | 973 | 140 | 40,5 |
| 78 | AH | 40 | 40 | 0 | 0 | 3 | 1 | 2 | 0 | 100 | 100 | 0 | 0 | 27,2 | 0,25 | 0,71 | 23,6 | 5,7 | 0,31 |
| 79 | AH | 0 | 0 | 0 | 0 | 0 | 0 | 0 | 0 | 100 | 100 | 0 | 0 | 1344 | 18,8 | 80,4 | 1150 | 168 | 61,5 |
| 80 | AH | 0 | 0 | 0 | 0 | 20 | 10 | 10 | 0 | 100 | 100 | 0 | 0 | 1271 | 20,6 | 28 | 2080 | 216 | 42,3 |
| 81 | AH | 40 | 40 | 0 | 0 | 10 | 5 | 5 | 0 | 100 | 50 | 50 | 0 | 1750 | 30,6 | 108,2 | 1432 | 292 | 93,3 |
| 82 | AH | 0 | 0 | 0 | 0 | 0 | 0 | 0 | 0 | 40 | 40 | 0 | 0 | 857 | 10,3 | 46,1 | 821 | 139,5 | 39 |
| 83 | AH | 10 | 10 | 0 | 0 | 5 | 5 | 0 | 0 | 90 | 90 | 0 | 0 | 1433 | 22,2 | 21,7 | 1879 | 345 | 22,1 |
| 84 | AH | 0 | 0 | 0 | 0 | 10 | 5 | 5 | 0 | 100 | 100 | 0 | 0 | 32,6 | 0,86 | 0,87 | 34,4 | 9 | 1,7 |
| 85 | AH | 0 | 0 | 0 | 0 | 5 | 2 | 3 | 0 | 100 | 80 | 20 | 0 | 1347 | 30,1 | 24,6 | 1717 | 195 | 24,4 |
| 86 | AH | 0 | 0 | 0 | 0 | 0 | 0 | 0 | 0 | 100 | 100 | 0 | 0 | 1261 | 18,7 | 48,4 | 1148 | 212 | 47 |
| 87 | AH | 0 | 0 | 0 | 0 | 1 | 0 | 0 | 0 | 90 | 90 | 0 | 0 | 1383 | 24,8 | 45,5 | 1167 | 186 | 42 |
| 88 | PC | 0 | 0 | 0 | 0 | 5 | 5 | 0 | 0 | 90 | 90 | 0 | 0 | NULL | NULL | NULL | NULL | NULL | NULL |
| 89 | PC | 0 | 0 | 0 | 0 | 30 | 20 | 10 | 0 | 100 | 20 | 80 | 0 | NULL | NULL | NULL | NULL | NULL | NULL |
| 90 | PC | 0 | 0 | 0 | 0 | 30 | 20 | 10 | 0 | 30 | 30 | 0 | 0 | NULL | NULL | NULL | NULL | NULL | NULL |
| 91 | PC | 0 | 0 | 0 | 0 | 0 | 0 | 0 | 0 | 30 | 30 | 0 | 0 | NULL | NULL | NULL | NULL | NULL | NULL |
| 92 | PC | 10 | 10 | 0 | 0 | 20 | 10 | 10 | 0 | 100 | 100 | 0 | 0 | NULL | NULL | NULL | NULL | NULL | NULL |
| 93 | PC | 20 | 20 | 0 | 0 | 65 | 50 | 10 | 5 | 100 | 70 | 30 | 0 | NULL | NULL | NULL | NULL | NULL | NULL |
| 94 | PC | 50 | 35 | 10 | 5 | 30 | 20 | 10 | 0 | 100 | 0 | 100 | 0 | NULL | NULL | NULL | NULL | NULL | NULL |
| 95 | PC | 20 | 20 | 0 | 0 | 30 | 20 | 5 | 5 | 100 | 100 | 0 | 0 | NULL | NULL | NULL | NULL | NULL | NULL |
| 96 | PC | 20 | 20 | 0 | 0 | 80 | 50 | 20 | 10 | 100 | 60 | 40 | 0 | NULL | NULL | NULL | NULL | NULL | NULL |
| 97 | PC | 70 | 50 | 20 | 0 | 80 | 50 | 20 | 10 | 100 | 60 | 40 | 0 | NULL | NULL | NULL | NULL | NULL | NULL |
| 98 | PC | 40 | 30 | 10 | 0 | 30 | 20 | 5 | 5 | 100 | 70 | 30 | 0 | NULL | NULL | NULL | NULL | NULL | NULL |
| 99 | PC | 0 | 0 | 0 | 0 | 95 | 50 | 30 | 15 | 100 | 0 | 100 | 0 | NULL | NULL | NULL | NULL | NULL | NULL |
| 100 | PC | 0 | 0 | 0 | 0 | 40 | 25 | 10 | 5 | 100 | 70 | 30 | 0 | NULL | NULL | NULL | NULL | NULL | NULL |
| 101 | PC | 0 | 0 | 0 | 0 | 8 | 5 | 2 | 1 | 100 | 70 | 30 | 0 | NULL | NULL | NULL | NULL | NULL | NULL |
| 102 | PC | 80 | 40 | 40 | 0 | 60 | 55 | 5 | 0 | 100 | 0 | 20 | 80 | NULL | NULL | NULL | NULL | NULL | NULL |
| 103 | PC | 50 | 50 | 0 | 0 | 30 | 25 | 5 | 0 | 100 | 0 | 100 | 0 | NULL | NULL | NULL | NULL | NULL | NULL |
| 104 | PC | 50 | 50 | 0 | 0 | 30 | 20 | 10 | 0 | 100 | 0 | 100 | 0 | NULL | NULL | NULL | NULL | NULL | NULL |
| 105 | PC | 60 | 50 | 10 | 0 | 75 | 50 | 20 | 5 | 100 | 0 | 100 | 0 | NULL | NULL | NULL | NULL | NULL | NULL |
| 106 | PC | 0 | 0 | 0 | 0 | 12 | 10 | 2 | 0 | 100 | 0 | 100 | 0 | NULL | NULL | NULL | NULL | NULL | NULL |
| 107 | PC | 20 | 20 | 0 | 0 | 30 | 20 | 10 | 0 | 100 | 50 | 50 | 0 | NULL | NULL | NULL | NULL | NULL | NULL |
| 108 | PC | 70 | 60 | 10 | 0 | 40 | 20 | 10 | 10 | 100 | 0 | 100 | 0 | NULL | NULL | NULL | NULL | NULL | NULL |
| 109 | PC | 10 | 10 | 0 | 0 | 55 | 40 | 15 | 0 | 100 | 100 | 0 | 0 | NULL | NULL | NULL | NULL | NULL | NULL |
| 110 | PC | 80 | 50 | 30 | 0 | 10 | 10 | 0 | 0 | 100 | 100 | 0 | 0 | NULL | NULL | NULL | NULL | NULL | NULL |
| 111 | PC | 0 | 0 | 0 | 0 | 5 | 5 | 0 | 0 | 100 | 100 | 0 | 0 | NULL | NULL | NULL | NULL | NULL | NULL |
| 112 | PC | 10 | 10 | 0 | 0 | 0 | 0 | 0 | 0 | 100 | 0 | 0 | 100 | NULL | NULL | NULL | NULL | NULL | NULL |
| 113 | PC | 10 | 10 | 0 | 0 | 40 | 30 | 10 | 0 | 100 | 40 | 60 | 0 | NULL | NULL | NULL | NULL | NULL | NULL |
| 114 | PC | 60 | 50 | 10 | 0 | 60 | 30 | 20 | 10 | 100 | 20 | 80 | 0 | NULL | NULL | NULL | NULL | NULL | NULL |
| 115 | PC | 15 | 10 | 0 | 5 | 15 | 10 | 5 | 0 | 100 | 70 | 30 | 0 | NULL | NULL | NULL | NULL | NULL | NULL |
| 116 | PC | 20 | 20 | 0 | 0 | 30 | 20 | 10 | 0 | 100 | 40 | 60 | 0 | NULL | NULL | NULL | NULL | NULL | NULL |
| 117 | PC | 0 | 0 | 0 | 0 | 30 | 10 | 10 | 10 | 100 | 80 | 20 | 0 | NULL | NULL | NULL | NULL | NULL | NULL |
| 118 | PC | 0 | 0 | 0 | 0 | 10 | 10 | 10 | 0 | 100 | 20 | 80 | 0 | NULL | NULL | NULL | NULL | NULL | NULL |
| 119 | PC | 0 | 0 | 0 | 0 | 35 | 20 | 10 | 5 | 100 | 0 | 70 | 30 | NULL | NULL | NULL | NULL | NULL | NULL |
