# Supplementary material for: HNF1B, EZH2 and ECI2 in prostate carcinoma. Molecular, immunohistochemical and clinico-pathological study
Source: Sci Rep. 2020 Sep 1;10:14365. doi: 10.1038/s41598-020-71427-7 (PMC7463257; doi:10.1038/s41598-020-71427-7)
Supplement: Supplementary file 2 — Supplementary Table 2. [file 41598_2020_71427_MOESM2_ESM.docx]

HNF1B, EZH2 and ECI2 in prostate carcinoma. Molecular, immunohistochemical and clinico-pathological study.

Running title: HNF1B, EZH2 and ECI2 in prostate carcinoma

Pavel Dundr^1*^, Michaela Bártů^1^, Jan Hojný^1^, Romana Michálková^1^, Nikola Hájková^1^, Ivana Stružinská^1^, Eva Krkavcová^1^, Ladislav Hadravský^2^, Lenka Kleissnerová^1^, Jana Kopejsková^1^, Bui Quang Hiep^1^, Kristýna Němejcová^1^, Radek Jakša^1^, Otakar Čapoun^3^, Jakub Řezáč^3^, Kateřina Jirsová^4^, Věra Franková^5^

^1^Institute of Pathology, First Faculty of Medicine, Charles University and General University Hospital in Prague, Czech Republic

^2^Institute of Pathology, First Faculty of Medicine, Charles University, Czech Republic

^3^Department of Urology, First Faculty of Medicine, Charles University and General University Hospital in Prague, Czech Republic

^4^Institute of Biology and Medical Genetics, First Faculty of Medicine, Charles University and General University Hospital in Prague, Czech Republic

^5^Department of Pediatrics and Adolescent Medicine, First Faculty of Medicine, Charles University and General University Hospital in Prague, Czech Republic

*Corresponding author:

Pavel Dundr, M.D., Ph.D.

Institute of Pathology, First Faculty of Medicine, Charles University and General University Hospital in Prague, Studničkova 2, 12800 Prague 2, Czech Republic

Email: [pavel.dundr@vfn.cz](mailto:pavel.dundr@vfn.cz)

Supplementary Table S2: Association of mRNA expression of HNF1B/EZH2/ECI2 and clinico-pathological variables, based on 491 patients with prostate carcinoma from TCGA atlas. Significant p-values are indicated in bold.

| Characteristic | Group | N | HNF1B mRNA mean | HNF1B mRNA median | *p-value* | EZH2 mRNA mean | EZH2 mRNA median | *p-value* | ECI2 mRNA mean | ECI2 mRNA median | *p-value* |
| --- | --- | --- | --- | --- | --- | --- | --- | --- | --- | --- | --- |
| Age |  |  |  |  | **0.045** |  |  | **0.002** |  |  | 0.858 |
| (mean=61, median=61) | <61 | 222 | 0.040 | -0.135 |  | -0.185 | -0.308 |  | -0.023 | -0.135 |  |
|  | ≥61 | 269 | -0.080 | -0.313 |  | 0.120 | -0.186 |  | 0.007 | -0.170 |  |
| T stage |  |  |  |  | 0.103 |  |  | **< 0.001** |  |  | 0.513 |
|  | T1 | 177 | -0.053 | -0.163 |  | -0.212 | -0.391 |  | 0.053 | -0.150 |  |
|  | T2 | 170 | 0.040 | -0.178 |  | -0.068 | -0.189 |  | -0.042 | -0.182 |  |
|  | T3+4 | 53 | -0.174 | -0.529 |  | 0.491 | 0.103 |  | 0.202 | -0.087 |  |
| Gleason score | |  |  |  | **< 0.001** |  |  | **< 0.001** |  |  | 0.666 |
|  | 1 | 45 | 0.246 | 0.005 |  | -0.411 | -0.434 |  | -0.099 | -0.125 |  |
|  | 2 | 145 | 0.099 | -0.013 |  | -0.391 | -0.434 |  | -0.126 | -0.253 |  |
|  | 3 | 99 | 0.036 | -0.092 |  | -0.136 | -0.271 |  | 0.048 | -0.089 |  |
|  | 4 | 63 | 0.036 | -0.233 |  | 0.196 | -0.029 |  | 0.071 | -0.147 |  |
|  | 5 | 139 | -0.315 | -0.711 |  | 0.487 | 0.155 |  | 0.073 | -0.159 |  |
| Methylation |  |  |  |  | **< 0.001** |  |  |  |  |  |  |
|  | Yes | 231 | -0.617 | -0.810 |  |  |  |  |  |  |  |
|  | No | 260 | 0.503 | 0.268 |  |  |  |  |  |  |  |
