# Supplementary material for: HNF1B, EZH2 and ECI2 in prostate carcinoma. Molecular, immunohistochemical and clinico-pathological study
Source: Sci Rep. 2020 Sep 1;10:14365. doi: 10.1038/s41598-020-71427-7 (PMC7463257; doi:10.1038/s41598-020-71427-7)
Supplement: Supplementary file 3 — Supplementary Table 3. [file 41598_2020_71427_MOESM3_ESM.docx]

HNF1B, EZH2 and ECI2 in prostate carcinoma. Molecular, immunohistochemical and clinico-pathological study.

Running title: HNF1B, EZH2 and ECI2 in prostate carcinoma

Pavel Dundr^1*^, Michaela Bártů^1^, Jan Hojný^1^, Romana Michálková^1^, Nikola Hájková^1^, Ivana Stružinská^1^, Eva Krkavcová^1^, Ladislav Hadravský^2^, Lenka Kleissnerová^1^, Jana Kopejsková^1^, Bui Quang Hiep^1^, Kristýna Němejcová^1^, Radek Jakša^1^, Otakar Čapoun^3^, Jakub Řezáč^3^, Kateřina Jirsová^4^, Věra Franková^5^

^1^Institute of Pathology, First Faculty of Medicine, Charles University and General University Hospital in Prague, Czech Republic

^2^Institute of Pathology, First Faculty of Medicine, Charles University, Czech Republic

^3^Department of Urology, First Faculty of Medicine, Charles University and General University Hospital in Prague, Czech Republic

^4^Institute of Biology and Medical Genetics, First Faculty of Medicine, Charles University and General University Hospital in Prague, Czech Republic

^5^Department of Pediatrics and Adolescent Medicine, First Faculty of Medicine, Charles University and General University Hospital in Prague, Czech Republic

*Corresponding author:

Pavel Dundr, M.D., Ph.D.

Institute of Pathology, First Faculty of Medicine, Charles University and General University Hospital in Prague, Studničkova 2, 12800 Prague 2, Czech Republic

Email: [pavel.dundr@vfn.cz](mailto:pavel.dundr@vfn.cz)

Supplementary table 3: List of primers

| **List of gene-specific primers used for *HNF1B* mutation analysis with universal adaptor sequences (red) by amplicon NGS** | | | | | | |
| --- | --- | --- | --- | --- | --- | --- |
| Amplicon number | Amplicon length (bp) | Primer Name | Full primer sequence (5' → 3') | Primer length (bp; without universal sequence) | % GC (without universal sequence) | Tm (without universal sequence) |
| 1 | 276 | HNF1B_NGS_e1F1 | ACACTCTTTCCCTACACGACGCTCTTCCGATCTGTTAGAAGTTTTCTGACTCCTTTCG | 25 | 40,0 | 59,1 |
|  |  | HNF1B_NGS_e1R1 | GTGACTGGAGTTCAGACGTGTGCTCTTCCGATCTAACTCCTCCAAGGCCTGAAC | 20 | 55,0 | 60,6 |
| 2 | 279 | HNF1B_NGS_e1F2 | ACACTCTTTCCCTACACGACGCTCTTCCGATCTGAAAATGGTGTCCAAGCTCAC | 21 | 47,6 | 59,6 |
|  |  | HNF1B_NGS_e1R2 | GTGACTGGAGTTCAGACGTGTGCTCTTCCGATCTAGGATGGGAGGTGTGTCATAGTC | 23 | 52,2 | 61,5 |
| 3 | 270 | HNF1B_NGS_e1F3 | ACACTCTTTCCCTACACGACGCTCTTCCGATCTCAAGCCGGTCTTCCATACTCT | 21 | 52,4 | 60,6 |
|  |  | HNF1B_NGS_e1R3 | GTGACTGGAGTTCAGACGTGTGCTCTTCCGATCTAACGGGCTTGGCGAGTGT | 18 | 61,1 | 64,1 |
| 4 | 247 | HNF1B_NGS_e2F | ACACTCTTTCCCTACACGACGCTCTTCCGATCTTAACCATCTGCTTGTCTGTCTAGTG | 25 | 44,0 | 59,9 |
|  |  | HNF1B_NGS_e2R | GTGACTGGAGTTCAGACGTGTGCTCTTCCGATCTCAGAGGCAGGATGAAAACACTTAC | 24 | 45,8 | 61,4 |
| 5 | 276 | HNF1B_NGS_e3F1 | ACACTCTTTCCCTACACGACGCTCTTCCGATCTGTCTGTCTGTCTGTCTGTCTGCTG | 24 | 54,2 | 61,6 |
|  |  | HNF1B_NGS_e3R1 | GTGACTGGAGTTCAGACGTGTGCTCTTCCGATCTGTAGGCCTGGTACAAGATTTGC | 22 | 50,0 | 60,0 |
| 6 | 292 | HNF1B_NGS_e3F2 | ACACTCTTTCCCTACACGACGCTCTTCCGATCTCTGTTTCTCTTTCCAGAGTTCAGTC | 25 | 44,0 | 60,0 |
|  |  | HNF1B_NGS_e3R2 | GTGACTGGAGTTCAGACGTGTGCTCTTCCGATCTACTAGTGTCTCAATATCCCAGGACC | 25 | 48,0 | 61,0 |
| 7 | 264 | HNF1B_NGS_e4F1 | ACACTCTTTCCCTACACGACGCTCTTCCGATCTGACTGCTGTGATTGTGTGTTTTTG | 24 | 41,7 | 61,9 |
|  |  | HNF1B_NGS_e4R1 | GTGACTGGAGTTCAGACGTGTGCTCTTCCGATCTAGAGGGTTCAGGCTGTGAGTC | 21 | 57,1 | 60,8 |
| 8 | 253 | HNF1B_NGS_e4F2 | ACACTCTTTCCCTACACGACGCTCTTCCGATCTCACTGAGGTCCGTGTCTACAAC | 22 | 54,5 | 59,7 |
|  |  | HNF1B_NGS_e4R2 | GTGACTGGAGTTCAGACGTGTGCTCTTCCGATCTAACCCTTAAACCAGATAAGATCCG | 24 | 41,7 | 60,9 |
| 9 | 251 | HNF1B_NGS_e5F | ACACTCTTTCCCTACACGACGCTCTTCCGATCTACTAATGTTCCCTACTGGGTTTGTG | 25 | 44,0 | 61,6 |
|  |  | HNF1B_NGS_e5R | GTGACTGGAGTTCAGACGTGTGCTCTTCCGATCTGTTTTGCCTCTTATCTTATCAGCTC | 25 | 40,0 | 59,1 |
| 10 | 272 | HNF1B_NGS_e6F | ACACTCTTTCCCTACACGACGCTCTTCCGATCTATCGCTAAGTCACATCGTGTTG | 22 | 45,5 | 60,1 |
|  |  | HNF1B_NGS_e6R | GTGACTGGAGTTCAGACGTGTGCTCTTCCGATCTTGCTTCCCATTCTTCTTCTCCC | 22 | 50,0 | 61,0 |
| 11 | 295 | HNF1B_NGS_e7F | ACACTCTTTCCCTACACGACGCTCTTCCGATCTCACCTCTCCTTATCCCAGGAG | 21 | 57,0 | 58,2 |
|  |  | HNF1B_NGS_e7R | GTGACTGGAGTTCAGACGTGTGCTCTTCCGATCTACTGAGGGTCCTGAGTGC | 18 | 61,0 | 60,4 |
| 12 | 247 | HNF1B_NGS_e8F | ACACTCTTTCCCTACACGACGCTCTTCCGATCTTACCTGTGTCTTTGCCTGTGTATG | 24 | 45,8 | 61,3 |
|  |  | HNF1B_NGS_e8R | GTGACTGGAGTTCAGACGTGTGCTCTTCCGATCTAACCTCTGCACATCCATGG | 19 | 52,6 | 58,7 |
| 13 | 285 | HNF1B_NGS_e9F | ACACTCTTTCCCTACACGACGCTCTTCCGATCTAGTTGGGCATCATCTCCCTTAG | 22 | 50,0 | 61,7 |
|  |  | HNF1B_NGS_e9R | GTGACTGGAGTTCAGACGTGTGCTCTTCCGATCTCTCCTGAGAGTGGATTGTCTGAG | 23 | 52,2 | 60,4 |
| 14 | 269 | HNF1B_chr17_rs4430796_F | ACACTCTTTCCCTACACGACGCTCTTCCGATCTGAGCGAGAGACCTTTCCAAG | 20 | 55,0 | 60,5 |
|  |  | HNF1B_chr17_rs4430796_R | GTGACTGGAGTTCAGACGTGTGCTCTTCCGATCTTTGAGAAGTTTCCAGTCTGCC | 21 | 47,6 | 59,8 |
| 15 | 250 | HNF1B_chr17_rs757210_F | ACACTCTTTCCCTACACGACGCTCTTCCGATCTGTCACCTCCACACAAGTCAG | 20 | 55,0 | 61,1 |
|  |  | HNF1B_chr17_rs757210_R | GTGACTGGAGTTCAGACGTGTGCTCTTCCGATCTCATCTAATTCTTACGACCGCTCC | 23 | 47,8 | 60,4 |
|  |  |  |  |  |  |  |
| **Primer pair used for Sanger sequencing of *HNF1B* 5' part of low-covered exon 4** | | | | | | |
| Amplicon number | Amplicon length (bp) | Primer Name | Full primer sequence (5' → 3') | Primer length (bp) | % GC | Tm |
| 1 | 232 | HNF1B e4F | TTGGCCAAGCACCAACAAGTC | 21 | 52,4 | 58,2 |
|  |  | HNF1B e4R1 | CTATAGCTCCAACCAGACTCACAGC | 25 | 52,0 | 58,5 |
|  |  |  |  |  |  |  |
| **Primer pair used for methylation analysis of *HNF1B* promoter** | | | | | | |
| Amplicon number | Amplicon length (bp) | Primer Name | Full primer sequence (5' → 3') | Primer length (bp) | % GC | Tm |
| 1 | 256 | HNF1B_mp3F | TTTTTGGATTTGTTAAGTTAGTGTTTT | 27 | 22,2 | 58,0 |
|  |  | HNF1B_mp3R | CCCTTCCTAAATAATCAATTTCTCTT | 26 | 30,8 | 59,7 |
|  |  |  |  |  |  |  |
| **Primer pairs used for ddPCR expression analysis of HNF1B, EZH2 and ECI2 mRNA targets** | | | | | | |
| Amplicon number | Amplicon length (bp) | Primer Name | Full primer sequence (5' → 3') | Primer length (bp) | % GC | Tm |
| 1 | 138 | ATP5F1B F (e4) | GCTCCCATTCATGCTGAGGC | 20 | 60,0 | 57,9 |
|  |  | ATP5F1B R (e5) | TCCAGCACCACCAAAAAGCC | 20 | 55,0 | 57,8 |
| 2 | 132 | HPRT1 F (e2) | GTGATGATGAACCAGGTTATGACCTTG | 27 | 44,4 | 57,7 |
|  |  | HPRT1 R (e3) | CTTCATCACATCTCGAGCAAGACG | 24 | 50,0 | 57,8 |
| 3 | 129 | POLR2A F (e27) | GCATGTTCTTTGGTTCAGCACC | 22 | 50,0 | 57,1 |
|  |  | POLR2A R (e28) | CCCCTGGGGTCATTCCACTC | 20 | 65,0 | 58,2 |
| 4 | 111 | HNF1B 5UTR F | CATGGCAAGTTAGAAGTTTTCTGACTCC | 28 | 42,9 | 58,1 |
|  |  | HNF1B 5UTR R | GCAAACCCCAAATCCAGGAACC | 22 | 54,5 | 58,4 |
| 5 | 117 | HNF1B 3UTR2 F | CTGCTGGCACCTCAGACAATC | 21 | 57,1 | 57,6 |
|  |  | HNF1B 3UTR2 R | CCAGGACAGACAGGAGTCCTTG | 22 | 59,1 | 58,1 |
| 6 | 121 | EZH2 F (e2) | CAAGAGGTTCAGACGAGCTGATG | 23 | 52,2 | 57,3 |
|  |  | EZH2 R (e3) | CACAGGCTGTATCCTTCGCTG | 21 | 57,1 | 57,4 |
| 7 | 180 | ECI2 F (e3) | GGCCACTGAAGGACCTTGTAAC | 22 | 54,5 | 57,3 |
|  |  | ECI2 R (e4) | CCAGGCTCCACCTGACTAGAG | 21 | 61,9 | 57,5 |
